# Wilms’ tumour gene 1 (WT1) enhances non-small cell lung cancer malignancy and is inhibited by microRNA-498-5p

**DOI:** 10.1186/s12885-023-11295-2

**Published:** 2023-09-04

**Authors:** Xuebing Li, Wenzhe An, Hongli Pan, Yaguang Fan, Hua Huang, Yixuan Wang, Wang Shen, Lingling Zu, Fanrong Meng, Xuexia Zhou

**Affiliations:** 1 Tianjin Key Laboratory of Lung Cancer Metastasis and Tumor Microenvironment, Tianjin Lung Cancer Institute, Department of Lung Cancer Surgery, Tianjin Medical University General Hospital, Tianjin, China; 2 Department of Neuropathology, Tianjin Key Laboratory of Injuries, Variations and Regeneration of the Nervous System, Key Laboratory of Post-trauma Neuro-repair and Regeneration in Central Nervous System of Education Ministry, Tianjin Neurological Institute, Tianjin Medical University General Hospital, Tianjin, China; 3 West China Hospital, Sichuan Lung Cancer Institute, Sichuan Lung Cancer Center, Sichuan University, Chengdu, China; 4 Tianjin Prenatal Diagnostic Center, Obstetrics and Gynecology Department, Tianjin Medical University General Hospital, Tianjin, China

**Keywords:** NSCLC, WT1, Malignancy, miR-498-5p, Prognostic biomarker

## Abstract

**Background:**

Wilms’ tumour gene 1 (WT1) is clearly recognized as a tumour promoter in diversiform of human malignancies. Nevertheless, knowledge of its expression, functions and potential molecular mechanisms in non-small cell lung cancer (NSCLC) remains elusive.

**Methods:**

Differential expression of WT1 mRNA and protein between NSCLC and normal tissues were assessed by analyzing RNA-seq data from Oncomine and protein data from Human Protein Atlas, respectively. Subsequently, prognosis significance and immune cell infiltration were analyzed by Kaplan-Meier plotter and CIBERSORT. 60 pairs of local NSCLC tissues were involved to validate WT1 expression by quantitative PCR (qPCR) and Western blot. Moreover, Cell Counting Kit-8 (CCK-8), colony formation, transwell, dual luciferase reporter assays and in vivo xenograft tumour growth experiments were conducted to explore the function and mechanism of WT1 in NSCLC.

**Results:**

Our solid data indicated that WT1 was increased in NSCLC tissues and cell lines in comparison with their matched controls. In particular, its upregulation correlated with worse prognosis and immune infiltration of the patients. Functional assays demonstrated that knockdown of WT1 inhibited NSCLC malignancy, including inhibiting cell proliferation, survival and invasion. Further exploration discovered that microRNA-498-5p (miR-498-5p) was the upstream suppressor of WT1 by directly targeting the 3’ untranslated region (UTR) of WT1 mRNA. Moreover, expression of miR-498-5p was notably decreased and inversely correlated with WT1 in NSCLC tissues. Finally, we proved that miR-498-5p was a potent tumour suppressor in NSCLC by suppressing cell proliferation, survival and invasion, while WT1 restoration could in turn disrupt this suppression both in vitro and in vivo.

**Conclusion:**

The abnormal increase in WT1 contributes to the malignant properties of NSCLC cells, and miR-498-5p is a natural inhibitor of WT1. Our findings might facilitate the development of novel therapeutic strategies against NSCLC in the future.

**Supplementary Information:**

The online version contains supplementary material available at 10.1186/s12885-023-11295-2.

## Introduction

Lung cancer now becomes the second most frequent human cancer and a dominant cause of cancer-associated death worldwide [1, 2]. Accompanied by high morbidity and mortality, lung cancer threatens public health, and increasing air pollution, especially in developing countries worsens the situation [3, 4]. Among all the cases of lung cancer, non-small cell lung cancer (NSCLC) takes the highest proportion (approximately 85%) [5]. Despite advances and improvements in diagnostic approaches and targeted therapies, the overall survival of lung cancer patients remains dismal, and NSCLC remains the most aggressive malignancy [5–7]. Organ metastasis is the major cause leading to the dismal outcome of NSCLC [8, 9]. Therefore, it is clinically desirable to develop novel biomarkers or therapeutic candidates to improve the early diagnosis and effective treatment of this disease.

Wilms’ tumour gene 1 (WT1) was first identified as a tumour suppressor in Wilms’ tumour, a childhood kidney neoplasm [10]. It belongs to the zinc finger transcription factor family with a DNA/RNA-binding domain in the C-terminal, and a glutamine/proline-rich domain in the N-terminal [11]. Emerging evidence characterizes WT1 as a tumour promoting factor in numerous human malignancies, including in leukaemia [12], breast cancer [13] and lung cancer [14]. Scholars recognize WT1 as a widespread tumour antigen, and recently, it ranks top in a list of 75 candidate cancer antigens [15]. For lung cancer, it has been shown that WT1 is overexpressed, and its high IgG antibody expression is associated with unsatisfactory patient outcome [16]. WT1 participates in the control of apoptosis, chemoresistance, proliferation and invasion of lung cancer cells via transcriptional modulation of its targets or interaction with the PI3K/AKT signalling pathway [14, 17, 18]. Herein, in this study, we conducted an integrated investigation of WT1 in vitro and in vivo, and the results strengthen its importance as an oncogene in lung carcinogenesis.

MicroRNAs (miRNAs) are a major type of endogenous short (17–25 nucleotides long) non-coding RNAs. They reduce their target expression by mRNA cleavage or translational repression [19]. They have emerged as star molecules during the last decades since their involvement in almost every aspect of cell biology. Moreover, abnormal expression of multiple miRNAs occurs in NSCLC, and they might serve as novel diagnostic or prognostic biomarkers [20, 21]. Through bioinformatics prediction, we identified miR-498-5p as an inhibitor of WT1. MiR-498-5p is a known tumour suppressor in NSCLC that targets high mobility group AT-hook 2 (HMGA2) [22].

In this study, we hypothesized that WT1 exerted tumour promoting effects in NSCLC and was targeted by miR-498-5p. We aimed to clarify the potential clinical significance, biological roles and upstream regulation of WT1 in NSCLC malignancy. Our findings indicate that WT1 might serve as a valuable prognostic biomarker and therapeutic target for NSCLC.

## Materials and methods

### Patients and tissue specimens

This study was approved by the ethical committee of Tianjin Medical University General Hospital (Tianjin, P. R. China). Sixty pairs of fresh NSCLC tissues with paired noncancerous lung tissues from NSCLC patients were collected after informed consent were obtained from all subjects. These patients were all diagnosed as NSCLC by histologically confirmation and did not receive any chemotherapy or radiotherapy before surgery. After resection, specimens were snap-frozen in liquid nitrogen and stored at − 80 °C until use.

### Cell culture and transfection

Normal control cell lines included Beas-2B (human bronchial epithelial cells) and MRC-5 (human embryonic lung fibroblast). NSCLC cell lines included A549, H460, H520, H661, H1299, YTMLC-90, SPC-A-1, 95 C, 95D, NL9980 and L9981. They were obtained from Cell Line Resource Center, Shanghai Institute of Biochemistry and Cell Biology, the Chinese Academy of Sciences (Shanghai, China) and cultured in F-12 K, DMEM or 1640 medium supplemented with 10% FBS (HyClone, USA) in a humidified atmosphere of 5% CO_2_ at 37 °C. The paired high-metastatic L9981 and low-metastatic NL9980 were established from a human lung large cell carcinoma cell line [23]. The paired high-metastatic 95D and low-metastatic 95 C were established from a human giant-cell lung carcinoma cell line [24]. Cell transfection was performed using Lipofectamine™ 2000 or Lipofectamine™ RNAiMAX Transfection Reagent (Life Technologies, USA) according to the standard instructions.

### MiRNA mimics, miRNA inhibitor and siRNAs

MiR-498-5p mimics, miR-498-5p inhibitor, WT1 siRNAs and their corresponding control oligonucleotides were ordered from GenePharma (Shanghai, P. R. China). Sequences of these oligonucleotides are presented in Table 1.

**Table 1:** Sequences and primers

Name	Sequence (5’-3’)
**Sequences of qPCR primers**	
*ACTB* real-time Forward	GATCATTGCTCCTCCTGAGC
*ACTB* real-time Reverse	ACTCCTGCTTGCTGATCCAC
*WT1* real-time Forward	CCACACCAGGACTCATACAGG
*WT1* real-time Reverse	GATGCATGTTGTGATGGCGG
**Sequences of cloning primers**	
WT1 clone no-tag Forward	CCGGAATTCCTGCAGGACCCGGCTTCC
WT1 clone no-tag Reverse	CCCGATATCTCAAAGCGCCAGCTGGAGT
WT1 3’-UTR WT Forward	CTAGCTAGCAAAAGAAAATAGGGGATG
WT1 3’-UTR WT Reverse	CCGCTCGAGTTGAGAGCAAAGTGAAAA
WT1 3’-UTR MT Forward	GGGAATTTATTATTTACCGTTCGAACTTTTTACTGTGTAAATATATGTC
WT1 3’-UTR MT Reverse	GACATATATTTACACAGTAAAAAGTTCGAACGGTAAATAATAAATTCCC
**Sequences of siRNAs**	
NC	UUCUCCGAACGUGUCACGUTT
siWT1-1	CCAAAUGACAUCCCAGCUUTT
siWT1-2	GGAGACAUACAGGUGUGAATT
**Sequences of miRNAs**	
miR-NC sence	UUCUCCGAACGUGUCACGUTT
miR-NC anti-sence	ACGUGACACGUUCGGAGAATT
miR-498-5p sence	UUUCAAGCCAGGGGGCGUUUUUC
miR-498-5p anti-sence	AAAACGCCCCCUGGCUUGAAAUU
anti-miR-NC	CAGUACUUUUGUGUAGUACAA
anti- miR-498-5p	GAAAAACGCCCCCUGGCUUGAAA

### Plasmid construction

The wild-type *WT1* 3’ UTR was inserted into the Dual-Luciferase miRNA Target Expression Vector pmirGLO (Promega, USA). The mutant *WT1* 3’ UTR reporter was constructed by site-directed mutagenesis on the basis of the wild-type *WT1* 3’ UTR reporter. WT1 coding sequence was cloned into the pcDNA3.1/Myc-His (+) A vector (Life Technologies, USA). Sequences for these cloning primers are presented in Table 1.

### RNA extraction and quantitative PCR (qPCR)

Total RNA was isolated from tissues or cells by TRIzol Reagent (Life Technologies, USA) according to standard procedures. Reverse transcription and qPCR were performed as per the standard protocol [25]. Briefly, 2 µg of RNA underwent reverse transcription using Reverse Transcriptase M-MLV (Promega, USA) to detect target mRNA/miRNA expression. qPCR was carried out on an Applied Biosystems 7900HT Fast Real-Time PCR System by using TB Green® Premix Ex Taq™ (Takara, Japan). β-actin or U6B was used as the housekeeping control for mRNA or miRNA, respectively. 2^–ΔΔCt^ method was applied to calculate the fold changes in mRNA or miRNA expression alterations. Primer sequences for qPCR are presented in Table 1. The qPCR primers for detecting miR-498-5p and U6B were ordered from RiboBio (Guangzhou, P. R. China).

### Protein extraction and western blot

Icecold RIPA lysis buffer (Beyotime Biotechnology, P. R. China) supplemented with phenylmethylsulfonyl fluoride was used to isolate total protein. Western blot was performed as per the standard protocol described previously [25]. In brief, 20 µg of protein was loaded and separated by 10% SDS-PAGE, then transferred onto a nitrocellulose membrane (Millipore Corporation, USA). Afterwards, the membrane was blocked with 5% skim milk for 1 h at room temperature and continuously incubated with the indicated primary antibodies at 4 °C overnight. Primary antibodies included mouse/rabbit anti-human WT1 (Abcam, USA), matrix metallopeptidase 2 (MMP2; Cell Signaling Technology, USA), c-myc (Santa Cruz Biotechnology, USA) and β-actin (Sigma-Aldrich Co., USA). After probed with horseradish peroxidase-linked secondary antibodies, the immune complexes on the membranes were detected by enhanced chemiluminescence Western Blotting Substrate (Pierce, USA). Quantification of protein expression by Western blot was completed using ImageJ software V1.8.0 (NIH, USA). Uncropped images for Western blots are provided in the Supplementary Material.

### Cell counting Kit-8 (CCK-8) assay

CCK-8 (Dojindo, Japan) assay was performed to evaluate cell proliferation ability as described previously [26]. Indicated A549 and H1299 cells were seeded into 96-well plates at a density of 1.2 × 10^3^/well, followed by incubation with the CCK-8 reagent for another 4 days. Each day, 10 µl WST®-8 from the CCK-8 kit was added to each well and incubated in the dark at 37 °C for another 2 h. Absorbance at the 450 nm wavelength was determined with a SpectraMax® M5 Multi-Mode Microplate Reader (Molecular Devices, USA).

### Colony formation assay

A colony formation assay was performed to reveal cell survival ability as described previously [27]. Briefly, indicated A549 and H1299 cells s were seeded into six-well plates at a density of 2 × 10^3^ cells per well. Every other day, the medium was changed. Fourteen days later, cells were fixed with 4% paraformaldehyde for 15 min, washed with PBS twice, and stained with 0.25% crystal violet solution for 30 min. After that, the cells were washed, dried in air and photographed. Then, 10% acetic acid was added into each well to dissolve the stained cells during shaking for 20 min. Absorbance at 600 nm was determined with a SpectraMax® M5 Multi-Mode Microplate Reader (Molecular Devices, USA).

### Transwell assay

Transwell assays were carried out to explore cell invasion ability as described previously [25]. Indicated A549 and H1299 cells were seeded into the upper chamber of Transwell® Permeable Supports (Corning Incorporated, USA) coated with Matrigel® Matrix (Corning Incorporated, USA) at a density of 2 × 10^3^/well in serum-free medium. Then, the cells were allowed to invade the bottom chamber containing medium supplemented with 10% FBS for 24 h. Invaded cells that reached the lower surface of the transwells were fixed with methanol and stained with 0.25% crystal violet. Non-invaded cells on the upper side were wiped with cotton swabs. The invaded cells were photographed under an inverted microscope (×400 magnification) and counted in at least five individual fields.

### Dual luciferase reporter assay

Dual luciferase reporter assays were done as described [28]. A549 and H1299 cells seeded at a density of 2 × 10^5^/well in 24-well plates were prepared before transfection. A total of 0.05 µg wild-type (WT) or mutant-type (MT) *WT1* 3’ UTR reporter was cotransfected either with controls or miR-498-5p mimics into cells. Forty-eight hours later, cells were harvested, and luciferase activities were detected using the Dual-Luciferase Reporter Assay System (Promega, USA). Firefly luciferase activity was normalized using the corresponding Renilla luciferase activity.

### Establishment of stable cell lines

To obtain a stable WT1-overexpressing cell line, cells transfected with WT1-expressing plasmid were selected by G418 (600 µg/ml) for two weeks. During these days, cell growth was monitored and fresh G418-containing medium was changed every other day. G418-resistant colonies were selected to form a pool, thus establishing the WT1-overexpressing stable cell line. To obtain a stable miR-498-5p-overexpressing cell line, cells were infected with lentiviruses expressing miR-498-5p or negative control (miR-NC) and selected by puromycin.

### In vivo xenograft tumour growth

In vivo xenograft tumour growth experiment was performed as described previously [29]. Male nude mice [BALB/c-nu (nu/nu)] at the age of four weeks were ordered from the Cancer Institute of the Chinese Academy of Medical Science (Beijing, P. R. China). Mice were maintained and used in accordance with the guidelines of the Institutional Animal Care and Use Committee of Tianjin Medical University General Hospital. A total of 5 × 10^6^ cells per mouse were subcutaneously injected into the flanks of the mice. Tumour volumes were measured every week with a caliper. Tumour volume was calculated by the equation: volume = a×b^2^ × 0.5326 (a is the longer dimension and b is the shorter dimension). Finally, the tumours in the mice were photographed and dissected, and the weights were measured.

### Statistical analysis

All graphs were plotted and statistically analyzed by GraphPad Prism V8.0. Data are expressed as the mean ± SD. Paired Student’s *t* test was performed to calculate the difference between expression levels in paired samples. Grouped Student’s *t* test or one-way ANOVA was performed to analyze the data from different groups. A chi-square test was performed to calculate the association between WT1 or miR-498-5p expression and clinicopathological parameters. The correlation between WT1 and miR-498-5p expression in NSCLC samples was analyzed by Spearman’s rank correlation. *P* < 0.05 was considered with statistical significance.

## Results

### Increased WT1 expression was associated with worse prognosis and immune infiltration of lung cancer patients

**Fig. 1:**
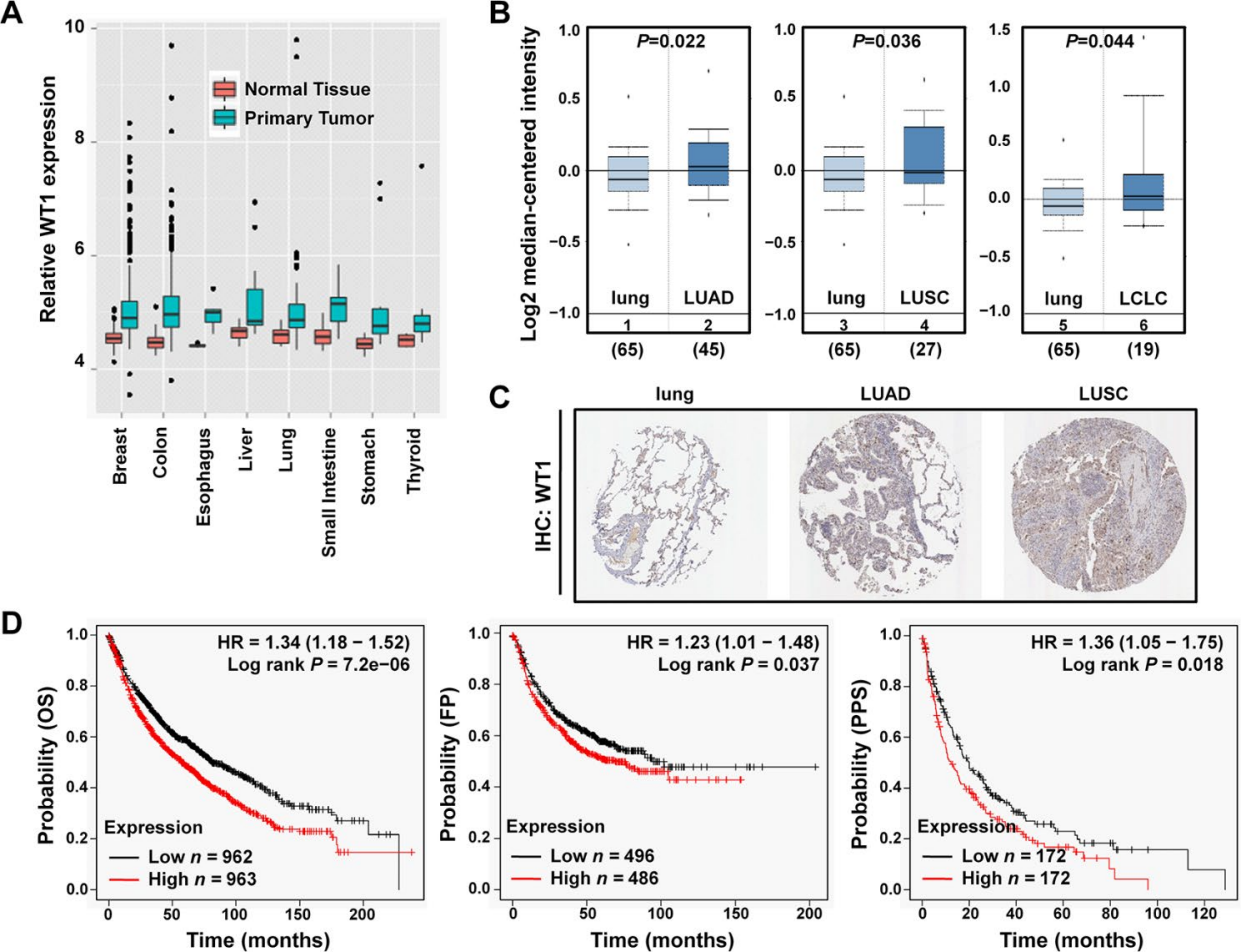
WT1 is highly expressed and its upregulation favors poor prognosis in lung cancer. **(A)** WT1 gene expression in different primary tumours and corresponding normal tissues by Metabolic gEne RApid Visualizer. **(B)** Comparison of WT1 gene expression in different subtypes of NSCLC tissues with normal lung tissues analyzed by Oncomine. Panels 1, 3, 5: normal lung tissues (n = 65). Panel 2: LUAD (n = 45). Panel 4: LUSC (n = 27). Panel 6: LCLC (n = 19). **(C)** WT1 protein expression in normal lung, LUAD and LUSC revealed by IHC from Human Protein Atlas. **(D)** Kaplan-Meier survival analysis of WT1 gene expression with NSCLC patient overall survival (OS), progression free survival (PFS) and post-progression survival (PPS) by Kaplan-Meier plotter

Based on the abovementioned knowledge of WT1, we hypothesized that WT1 acted as an oncogene in NSCLC and might be targeted by miRNAs. We performed comprehensive studies on its clinical significance, cellular functions and molecular mechanisms in NSCLC malignancy both in vitro and in vivo. To explicit WT1 involvement in carcinogenesis, we first evaluated its expression profile across various human cancers using the public Metabolic gEne RApid Visualizer database (http://merav.wi.mit.edu/). WT1 was increased in a majority of human primary tumours compared with the corresponding normal tissues, including lung cancer (Fig. 1A). Analyses of WT1 expression by Oncomine (http://www.oncomine.org) and Human Protein Atlas (https://www.proteinatlas.org/) indicated that WT1 was upregulated in lung adenocarcinoma (LUAD), lung squamous cell carcinoma (LUSC) and large cell lung carcinoma (LCLC) (Fig. 1B, C). Survival analyses by Kaplan-Meier plotter (http://kmplot.com/analysis/) indicated that higher WT1 expression predicted worse patient outcome (Fig. 1D). Therefore, the expression and clinical significance predicted by website tools all indicated that increased WT1 expression might contribute to lung cancer malignancy and poor patient prognosis.

**Fig. 2:**
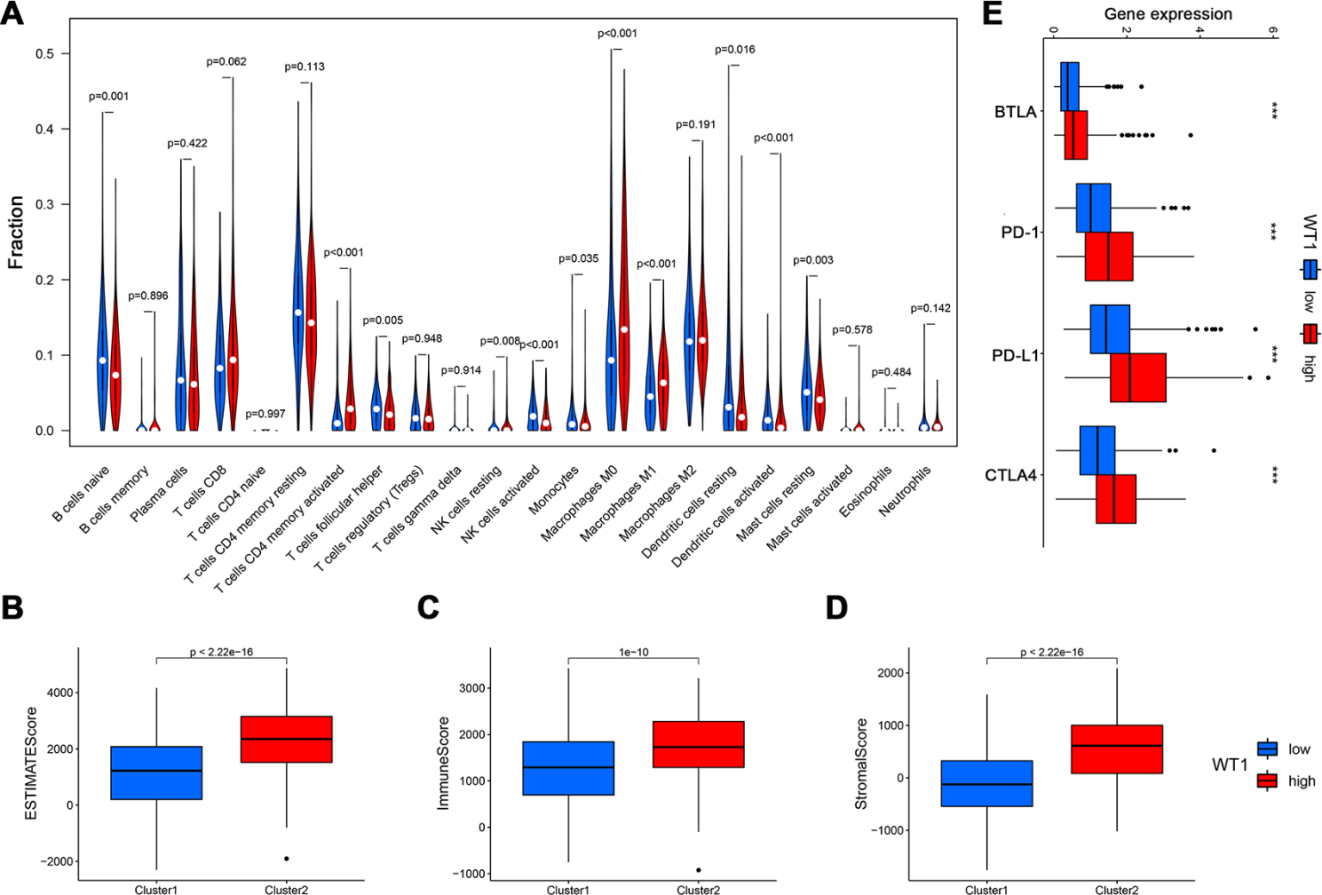
Immune microenvironment analyses between the high and low WT1 expression groups in the TCGA cohort. **(A)** Comparison of the difference of immune cell fractions between high and low WT1 expression groups via CIBERSORT method. **(B-D)** The differences of ESTIMATE scores, IMMUNE scores and Stromal Scores between high and low WT1 expression groups. **(E)** The differential expression of four common immune checkpoints between high and low WT1 expression groups. Wilcox test were used for data analysis

As an attractive tumour-associated antigen, WT1 is usually overexpressed in leukemia and various types of solid tumours. WT1-specific adoptive immunotherapy has been the “hot spot” for tumour treatment, which exhibit promising antineoplastic effect with tolerable safety [30]. However, its involvement in NSCLC immunotherapy remains very rare. For another, tumour infiltrating immune cells are important components of the tumour microenvironment and regulate tumour progression [31]. In order to further explore the relationship between WT1 and tumour immune microenvironment (TME), we calculated the immune infiltration abundance of patients in TCGA-LUAD cohort through CIBERSORT algorithm. CIBERSORT (https://cibersort.stanford.edu/) is an analysis tool to estimate the abundance of 22 immune member cell types, we divided patients into two groups according to the expression level of WT1, we found that tumour infiltration immune cells were significantly different between the two groups: B cells naive, T cells follicular helper, NK cells activated, monocytes, dendritic cells resting and mast cells resting were all significantly down-regulated in the high expression of the WT1 group. T cells CD4 memory activated, NK cells resting, macrophages M0 and macrophages M1 were all significantly up-regulated in the high expression of the WT1 group (Fig. 2A). We also calculated the estimate score, immune score and stromal score for each patient by the ESTIMATE algorithm. The results demonstrated that the estimate score, immune score and stromal score were higher in the WT1 high expression group (Fig. 2B-D). To further explore the differences in the response to immunotherapy between the two groups (high WT1 vs. low WT1), we compared the immune checkpoints expression. As shown in Fig. 2E, B- and T-lymphocyte attenuator (BTLA), programmed cell death (PD)-1, PD ligand 1 (PD-L1) and cytotoxic tlymphocyte-associated antigen (CTLA) were all elevated in the high WT1 group. These results further supported the impact of WT1 on TME immune activity.

### WT1 was upregulated in NSCLC and its higher expression correlated with advanced tumour grade

**Fig. 3:**
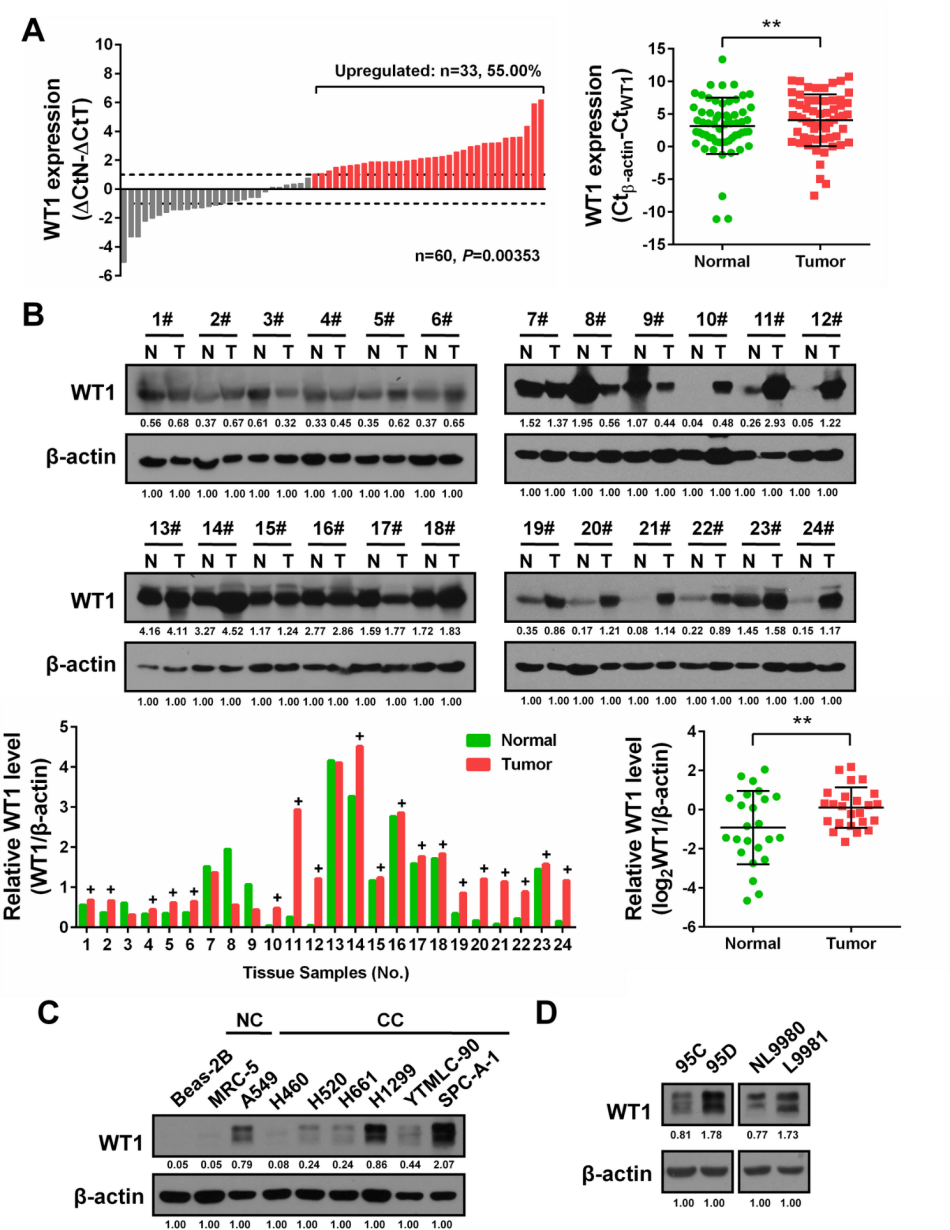
WT1 is overexpressed in NSCLC tissues and cell lines. **(A)** qPCR detection of WT1 mRNA expression in 60 pairs of NSCLC tissues. ΔCtN: threshold cycle (Ct) value of β-actin was subtracted from the Ct value of WT1 of paired normal tissue. ΔCtT: Ct value of β-actin was subtracted from that of WT1 of NSCLC tissue. Bar value (ΔCtN-ΔCtT) ≥ 1 or ≤ − 1 was considered to be significantly upregulation or downregulation, respectively. Red bars indicated WT1 upregulation (left panel). Comparison of WT1 mRNA expression between the same normal (green) and tumour (red) tissues (n = 60) was completed by calculating ΔCt_β−actin_-ΔCt_WT1_ (right panel). **P < 0.01. **(B)** Western blot (upper) and quantification (bottom) of WT1 expression in 24 pairs of NSCLC tissues. Loading control: β-actin. Ratio of WT1/β-actin was calculated and + indicated upregulation (bottom, left). Group comparison result was also provided (bottom, right). **P < 0.01. **(C)** Western blot of WT1 expression in the non-cancerous Beas-2B, MRC-5 cell lines and seven NSCLC cell lines. **(D)** Western blot of WT1 expression in two pairs of differential metastatic NSCLC cell lines (95 C vs. 95D, NL9980 vs. L9981)

To further confirm the elevated expression of WT1, we collected 60 pairs of NSCLC tissues with intact clinical pathology information from our hospital. qPCR and Western blot assays both indicated higher expression of WT1 in NSCLC tissues (Fig. 3A, B). Importantly, higher WT1 levels were obviously associated with advanced T/N stage and tumour grade (*P* < 0.05; Table 2). Its higher expression was also observed in NSCLC cell lines than in the normal Beas-2B and MRC-5 cell lines (Fig. 3C), and WT1 was extremely overexpressed in the high-metastatic 95D cells and L9981 cells compared with the corresponding low-metastatic 95 C cells and NL9980 cells, respectively (Fig. 3D). Taken together, these data strongly indicate that upregulation of WT1 might contribute to NSCLC development.

**Table 2:** Relationship between expression levels of WT1 and clinical and pathological features of the NSCLC individuals

Clinical Characteristics	Non-increased (ΔΔCt ≤ 1) n = 27	Increased (ΔΔCt > 1) n = 33	Test of Significance
**Sex**			
Female	9 (15.00%)	12 (20.00%)	χ^2^ = 0.0599
Male	18 (30.00%)	21 (35.00%)	*P* = 0.8066
**Age**			
< 60	15 (25.00%)	21 (35.00%)	χ^2^ = 0.4040
>=60	12 (20.00%)	12 (20.00%)	*P* = 0.5250
**Tumour Type**			
Squamous cell lung carcinoma	11 (18.33%)	14 (23.33%)	
Lung adenocarcinoma	16 (26.67%)	17 (28.33%)	χ^2^ = 1.8084
Others	0 (0.00%)	2 (3.33%)	*P* = 0.5825^ F^
**T Stage**			
T1	18 (30.00%)	9 (15.00%)	χ^2^ = 9.3113
T2 + T3	9 (15.00%)	24 (40.00%)	*P* = 0.0023
** N Stage**			
N0	18 (30.00%)	13 (21.67%)	χ^2^ = 4.4231
N1 + N2	9 (15.00%)	20 (33.33%)	*P* = 0.0355
**Tumour Grade**			
I + II	22 (36.67%)	17 (28.33%)	χ^2^ = 5.8615
III + IV	5 (8.33%)	16 (26.67%)	*P* = 0.0155
**Differentiation**			
Well	6 (10.00%)	8 (13.33%)	
Moderate	14 (23.33%)	19 (31.67%)	χ^2^ = 0.5255
Poor	7 (11.67%)	6 (10.00%)	*P* = 0.7689

F: Fisher’s exact test

### Network analysis of WT1 coexpressed genes in NSCLC

**Fig. 4:**
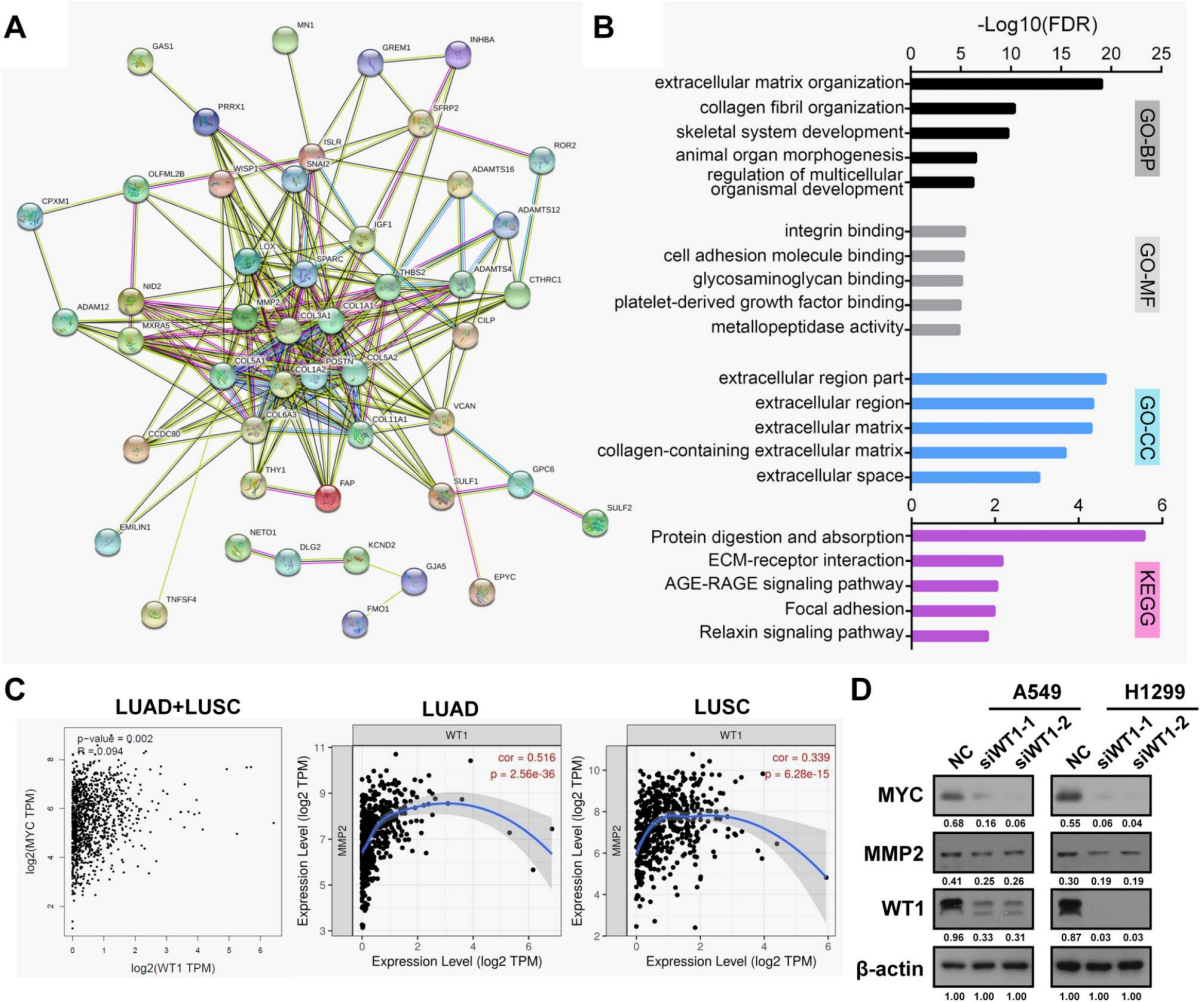
Network analysis of WT1 co-expressed genes indicates its participation in NSCLC progression. **(A)** Protein interaction network of WT1 co-expressed genes screened by cBioportal and plotted by STRING. **(B)** GO enrichment (BP: biological process, MF: molecular function, CC: cellular component) and KEGG pathway analyses of WT1 co-expressed genes. –Log_10_ transformed false discovery rates (FDR) were plotted for each enriched functional category. **(C)** Western blot examination of WT1, MYC and MMP2 after WT1 siRNA transfection in A549 and H1299 cells

To understand the general roles of WT1 in NSCLC cells, we filtered out WT1 coexpressed genes from the NSCLC gene expression profiling database in cBioPortal (https://www.cbioportal.org/) by setting the absolute value of Spearman’s correlation coefficient > 0.5. Then, 70 genes in total were submitted to the STRING database (https://cn.string-db.org/) to plot the gene interaction network (Fig. 4A) and predict the possible biological processes in which WT1 might be involved. Gene ontology (GO) analyses revealed that the coexpressed genes of WT1 were enriched in processes or functions such as extracellular matrix (ECM) organization and integrin binding (Fig. 4B). In addition, Kyoto Encyclopedia of Genes and Genomes (KEGG) pathway [32] analysis suggested possible roles of WT1 coexpressed genes in ECM-receptor interactions as well as focal adhesion (Fig. 4B). All these predicted roles of WT1 coexpressed genes were tightly linked with the cell motility and aggressiveness of cancer cells, strongly implying that WT1 might participate in NSCLC development at least via cell motility regulation.

Among these 70 coexpressed genes, we noticed two particular ones: MYC and MMP2, two well-recognized malignancy markers closely related to cancer cell proliferation, survival and invasion. MYC is a hub-gene in cancer progression by controlling almost every aspect of tumour malignancy. Previous reports have established that WT1 might activate MYC transcription in breast cancer and lung cancer cells [33, 34]. Besides, overexpression of matrix metalloproteinase (MMP) plays an important role in the context of tumour invasion and metastasis, and MMP2 has been characterized as the most validated target for cancer [35]. Therefore, we validated the impact of WT1 on MYC/MMP2 expression as priority. Expression correlation analyses of WT1 with MYC/MMP2 in LUAD and LUSC cases using the Gene Expression Profiling Interactive Analysis 2.0 (GEPIA2) portal (http://gepia2.cancer-pku.cn/) and Tumour Immune Estimation Resource 2.0 (TIMER2.0) portal (http://timer.cistrome.org/) indicated that WT1 levels were obviously correlated with MMP2/MYC levels (Fig. 4C). Furthermore, WT1 knockdown utilizing small interfering RNAs (siRNAs) resulted in the downregulation of MYC and MMP2 (Fig. 4D). Therefore, these coexpression analyses indicated that WT1 might be involved in NSCLC progression.

### WT1 exerted oncogenic activities in NSCLC cells

**Fig. 5:**
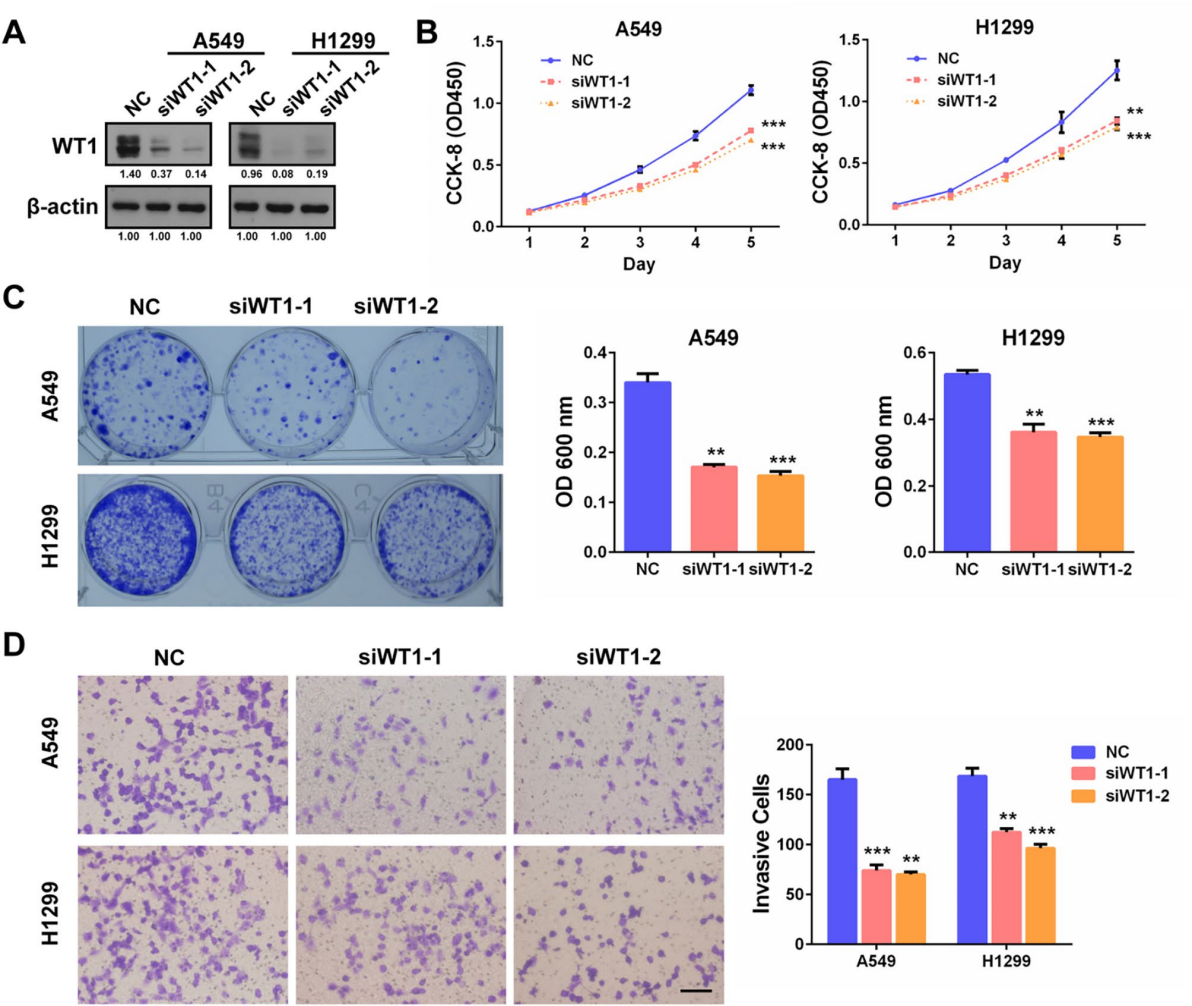
WT1 knockdown by siRNAs inhibits NSCLC cell proliferation, survival and invasion. **(A)** Western blot validation of WT1 knockdown by siRNA transfection inA549 and H1299 cells. **(B)** Growth curves of the indicated cells assessed by CCK-8 assays. **(C)** Colony formation results. Representative images (left) and quantification graph (right) were provided. **(D)** Transwell invasion results. Representative images (left) and quantification graph (right) were provided. **P < 0.01, ***P < 0.001. Scale bar, 200 μm

Based on the above findings, we then performed loss-of-function studies to confirm the functions of WT1 in NSCLC cells. A549 and H1299 cells were used since they expressed moderate endogenous WT1 levels (Fig. 3C). Western blot validated the efficient knockdown of WT1 by specific siRNAs in A549 and H1299 cells (Fig. 5A). Cell proliferation and survival abilities were determined by CCK-8 and colony formation assays, respectively. We observed that WT1 knockdown clearly inhibited cell proliferation and survival of the two cell lines compared with the negative control (NC) siRNA-treated groups (Fig. 5B, C**)**. Invasion capacity monitored by transwell assay was also significantly suppressed by WT1 knockdown (Fig. 5D). Collectively, these results clearly verified that WT1 is a proto-oncogene in NSCLC cells by facilitating cell proliferation, survival and invasion.

### WT1 is a direct target of miR-498-5p in NSCLC cells

**Fig. 6:**
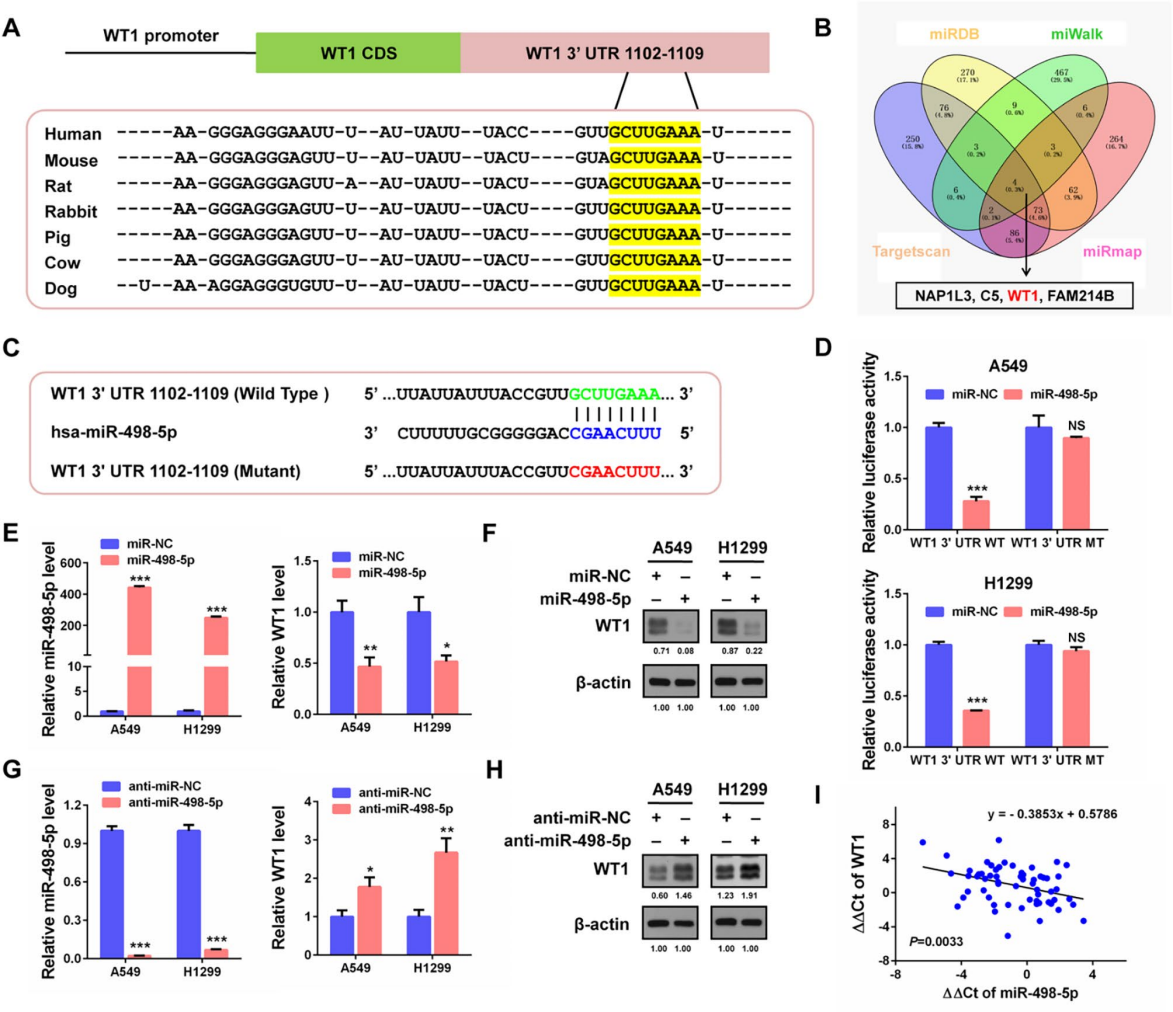
WT1 is a direct target of miR-498-5p and their expression inversely correlate with each other. **(A)** Schematic depicting of WT1 3’ UTR containing the conserved binding site of miR-498-5p (marked by a yellow background) across multiple species. **(B)** Target candidates of miR-498-5p were predicted using four bioinformatic tools: miRDB, miRwalk, Targetscan and miRmap. **(C)** Schematic diagram of constructing the wild type and mutant type WT 3’ UTR luciferase reporters. **(D)** Dual luciferase assay results in A549 and H1299 cells showing a direct targeting of WT1 by miR-498-5p. **(E)** qPCR detection of miR-498-5p and WT1 expression after transfection with miR-498-5p mimics. **(F)** Western blot detection of WT1 expression as transfected in (E). **(G)** qPCR detection of miR-498-5p and WT1 expression after transfection with miR-498-5p inhibitor (anti-miR-498-5p). **(H)** Western blot detection of WT1 expression as transfected in (G). **(I)** Correlation analysis of WT1 and miR-498-5p expression in 60 pairs of NSCLC tissues assessed by Spearman’s rank correlation analysis. *P < 0.05, **P < 0.01, ***P < 0.001. NS, no significant

To identify its natural inhibitor, namely, the potential upstream miRNA of WT1, we used TargetScan (https://www.targetscan.org/vert_80/) online software. As shown in Fig. 6A, the WT1 3’ UTR contained a putative miR-498-5p binding site with a high score, and this binding site showed high conservation across species, including humans (*Homo sapiens*), mice (*Mus musculus*), rats (*Rattus norvegicus*), rabbits (*Oryctolagus cuniculus*), pigs (*Sus scrofa*), cows (*Bos taurus*) and dogs (*Canis lupus familiaris*). Subsequent prediction of miR-498-5p targets using the four online bioinformatics resources miRDB (http://mirdb.org/), miWalk (http://mirwalk.umm.uni-heidelberg.de/), TargetScan and miRmap (https://mirmap.ezlab.org/) in combination showed that WT1 was among the four common genes (Fig. 6B), illustrating the reliability of miR-498-5p targeting WT1. Dual luciferase reporter assays proved the target relationship between miR-498-5p and WT1. The results revealed that transfection with miR-498-5p mimics clearly led to a reduction in the wild-type WT1 3’ UTR reporter activity, whereas they produced no effects on the mutant reporter (Fig. 6C, D). This finding suggested that WT1 was a direct target of miR-498-5p in NSCLC cells. qPCR and Western blot further verified that miR-498-5p significantly inhibited WT1 mRNA and protein expression in the two NSCLC cell lines (Fig. 6E, F). However, the miR-498-5p inhibitor (anti-miR-498-5p) exerted the opposite effects on WT1 expression (Fig. 6G, H). Furthermore, WT1 expression inversely correlated with miR-498-5p in NSCLC tissues (n = 60; Fig. 6I). Therefore, our results illustrate that miR-498-5p targets WT1 by binding to the target region of its 3’ UTR, and inhibits its expression via mRNA degradation induction.

**Table 3:** Relationship between expression levels of miR-498-5p and clinical and pathological features of the NSCLC individuals

Clinical Characteristics	Non-increased (ΔΔCt ≤ − 1) n = 28	Increased (ΔΔCt > − 1) n = 32	Test of Significance
**Sex**			
Female	13 (21.67%)	8 (13.33%)	χ^2^ = 3.0141
Male	15 (25.00%)	24 (40.00%)	*P* = 0.0825
**Age**			
< 60	18 (30.00%)	18 (30.00%)	χ^2^ = 0.4018
>=60	10 (16.67%)	14 (23.33%)	*P* = 0.5262
**Tumour Type**			
Squamous cell lung carcinoma	10 (16.67%)	15 (25.00%)	
Lung adenocarcinoma	16 (26.67%)	17 (28.33%)	χ^2^ = 2.7760
Others	2 (3.33%)	0 (0.00%)	*P* = 0.3037^ F^
**T Stage**			
T1	8 (13.33%)	19 (31.67%)	χ^2^ = 5.7251
T2 + T3	20 (33.33%)	13 (21.67%)	*P* = 0.0167
** N Stage**			
N0	9 (15.00%)	22 (36.66%)	χ^2^ = 8.0137
N1 + N2	19 (31.67%)	10 (16.67%)	*P* = 0.0046
**Tumour Grade**			
I + II	13 (21.67%)	26 (43.33%)	χ^2^ = 7.9592
III + IV	15 (25.00%)	6 (10.00%)	*P* = 0.0048
**Differentiation**			
Well	7 (11.67%)	7 (11.67%)	
Moderate	15 (25.00%)	18 (30.00%)	χ^2^ = 0.0834
Poor	6 (10.00%)	7 (11.67%)	*P* = 0.9592

F: Fisher’s exact test

### Downregulation of the tumour suppressive miR-498-5p in NSCLC

**Fig. 7:**
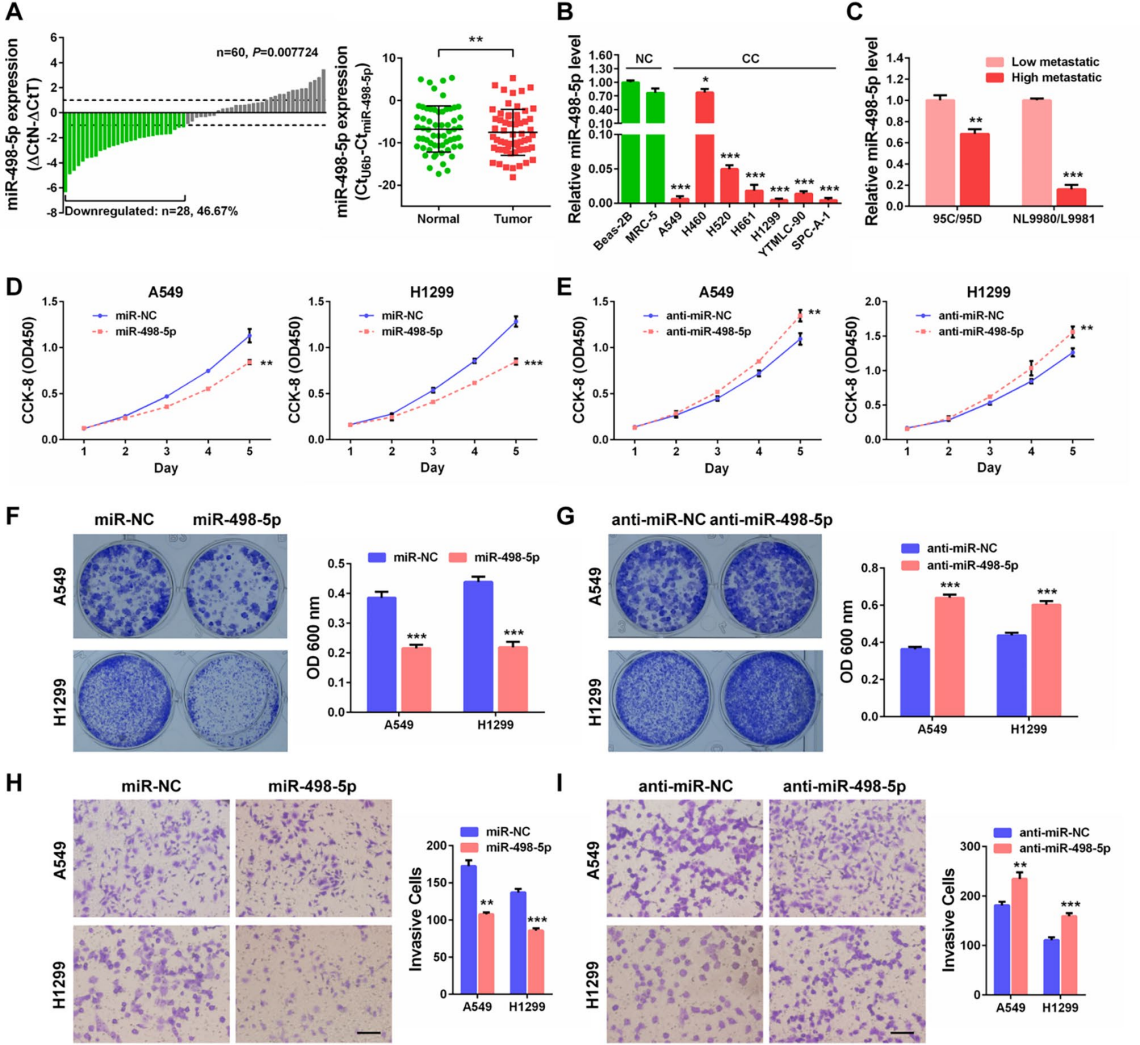
miR-498-5p is decreased and acts as a tumour suppressor by inhibiting cell proliferation, survival and invasion in NSCLC. **(A)** qPCR detection of miR-498-5p expression in 60 pairs of NSCLC tissues. Bar value (ΔCtN-ΔCtT) ≥ 1 or ≤ − 1 was considered to be significantly upregulation or downregulation, respectively. Green bars indicated miR-498-5p downregulation (left panel). Comparison of miR-498-5p expression between the same normal (green) and tumour (red) tissues (n = 60) was shown (right panel). **P < 0.01. **(B)** qPCR quantification of miR-498-5p expression in human non-cancerous Beas-2B, MRC-5 cell lines and seven NSCLC cell lines. **(C)** qPCR quantification of miR-498-5p expression in two pairs of differential metastatic NSCLC cell lines (95 C vs. 95D, NL9980 vs. L9981). **(D, E)** Growth curves of the indicated cells assessed by CCK-8 assays. **(F, G)** Colony formation results. Representative images (left) and quantification graph (right) were provided. **(H, I)** Transwell invasion results. Representative images (left) and quantification graph (right) were provided. **P < 0.01, ***P < 0.001. Scale bar, 200 μm

As proven above, WT1 exerted oncogenic activities by promoting NSCLC cell proliferation, survival and invasion, thus we suspected that its inhibitor, miR-498-5p, might possess the opposite tumour suppressive roles. MiR-498-5p expression in the abovementioned 60 pairs of NSCLC tissues was then determined by qPCR. The result revealed that miR-498-5p was downregulated in NSCLC tissues (Fig. 7A). Additionally, its decreased expression was also observed in NSCLC cell lines compared with the normal Beas-2B and MRC-5 cell lines (Fig. 7B), and extremely downregulated in high-metastatic 95D and L9981 cells compared with the corresponding low-metastatic 95 C and NL9980 cells, respectively (Fig. 7C). Furthermore, decreased miR-498-5p levels were markedly associated with advanced T/N stage and tumour grade (*P* < 0.05; Table 3). Consistently, NSCLC cells transfected with miR-498-5p mimics showed prominently reduced capacities for cell proliferation, survival and invasion (Fig. 7D, F, H), whereas cells transfected with its inhibitor possessed the opposite effects (Fig. 7E, G, I). Therefore, miR-498-5p perfectly simulated the suppressive roles of WT1 silencing on the malignancy of NSCLC cells. These findings indicate that downregulation of miR-498-5p is an important cause leading to WT1 overexpression in NSCLC and that miR-498-5p itself inhibits NSCLC progression.

#### WT1 restoration attenuated the tumour suppressing effects of miR-498-5p in NSCLC

**Fig. 8:**
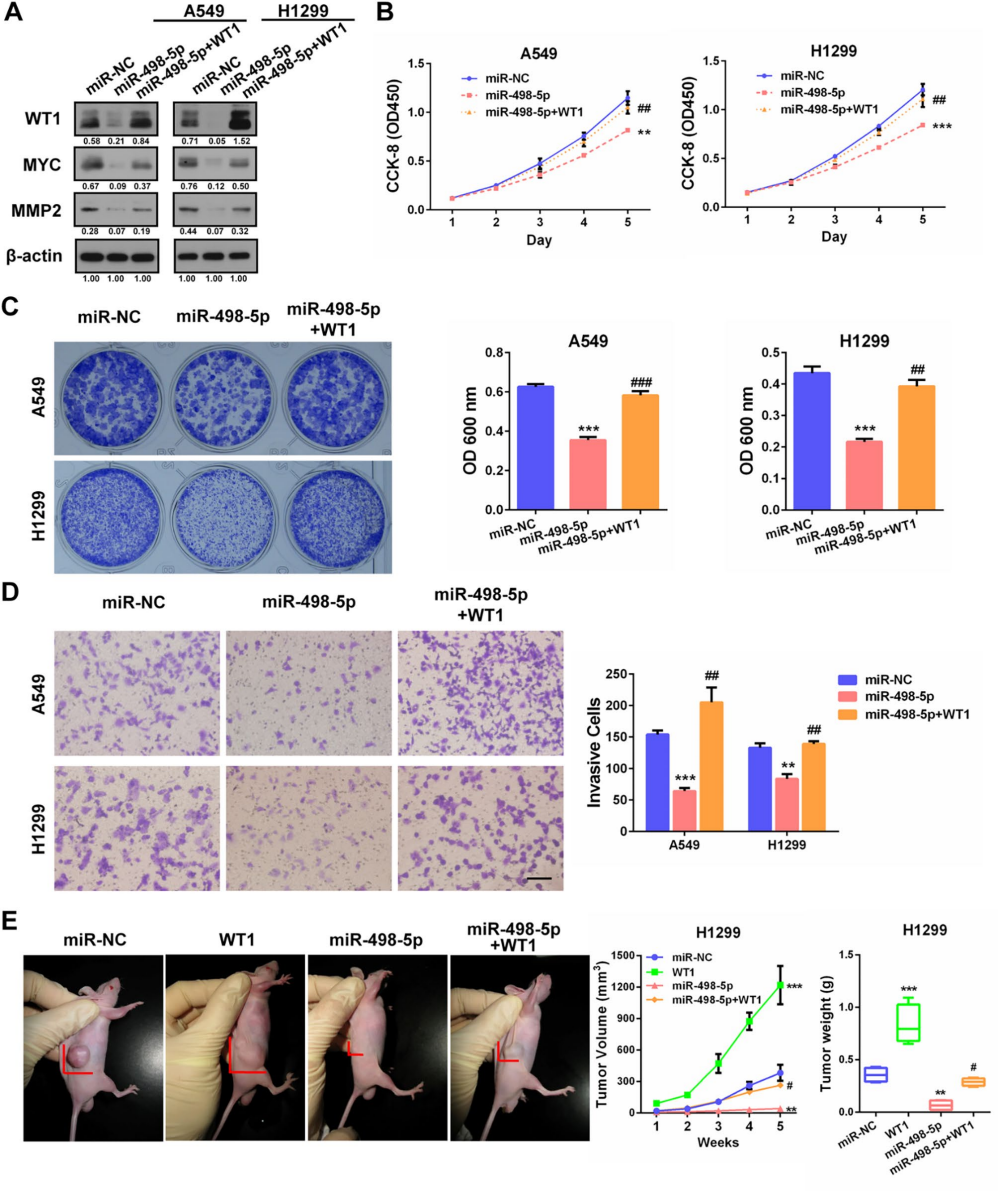
WT1 mediates the tumour suppressing effects of miR-498-5p in NSCLC. **(A)** Western blot of WT1, MYC, MMP2 after WT1 was restored in miR-498-5p-overexpressing A549 and H1299 cells. **(B)** Growth curves of the indicated cells assessed by CCK-8 assays. **(C)** Colony formation results. Representative images (left) and quantification graph (right) were provided. **(D)** Transwell invasion results. Representative images (left) and quantification graph (right) were provided. **(E)**In vivo xenograft tumour growth results of the indicated cells. Representative images (left), tumour volume (middle) and tumour weight (right) were provided. **P < 0.01, ***P < 0.001 (vs. miR-NC), ^#^*P* < 0.05, ^##^*P* < 0.01, ^###^*P* < 0.001 (miR-498-5p + WT1 vs. miR-498-5p). Scale bar, 200 μm

Finally, to determine whether miR-498-5p inhibited NSCLC malignancy through WT1, we restored WT1 expression in miR-498-5p-overexpressing cells by cotransfecting WT1 plasmid and miR-498-5p mimics. Western blot confirmed that WT1 plasmid cotransfection effectively restored the expression of WT1, MYC and MMP2 in A549 and H1299 cells (Fig. 8A). Subsequent functional assays showed that miR-498-5p observably impaired the proliferation, survival and invasion of A549 and H1299 cells, while WT1 restoration observably attenuated these inhibitory effects (Fig. 8B-D). In line with these in vitro results, xenografted tumours formed by miR-498-5p-overexpressing H1299 cells showed reduced tumorigenic potential compared with those formed by control cells, while WT1 overexpression showed enhanced tumour promoting capacity in nude mice. Moreover, WT1 recovery effectively relieved miR-498-5p-induced NSCLC inhibition in vivo (Fig. 8E). These results strongly suggest that miR-498-5p inhibits NSCLC malignancy by directly targeting WT1, a vital tumour promoter.

## Discussion

As a highly frequent and lethal human malignancy, lung cancer has posed a threat to public health for years. Among all lung cancers, NSCLC comprises a majority, and it includes several histological subgroups: LUAD, LUSC and LCLC. Herein, we focused on the expression, significance and biological roles of WT1 in NSCLC progression. Although some reports have described the function of WT1 in lung cancer over the past few decades, its impacts on the malignant properties (proliferation, migration, invasion and tumour formation) of NSCLC cells have not been fully elucidated. Herein, by utilizing online resources and performing loss-of-function experiments, we provided solid and comprehensive evidence to characterize the functions of WT1 in NSCLC.

Although originally considered as a tumour suppressor in Wilms’ tumour [10], subsequent studies in various types of human malignancies indicated that WT1 is overexpressed and possesses oncogenic activities via distinct mechanisms [12–18]. The involvement of WT1 in lung cancer has also been discussed previously: (1) overexpression of WT1 was demonstrated by qPCR and immunohistochemistry (IHC) in *de novo* NSCLC with no mutations [36]; (2) high level of WT1 IgG antibody expression might be a useful marker for the early detection of NSCLC and its prognostic prediction [16]; (3) WT1 deficiency impaired proliferation and induced senescence in lung cancer cells dependent on oncogenic KRAS signalling [37]; WT1 facilitates NSCLC cell proliferation by elevating cyclin D1 and p-pRb expression [17]; silencing of WT1 induced growth inhibition, cell cycle arrest and chemoresistance via the PI3K/AKT pathway in lung cancer cells [14].

Irrespective of the previous findings of WT1, we aimed to reconfirm the roles of WT1 in NSCLC. First, an online overview of its mRNA/protein expression across various types of human cancers revealed its frequent upregulation in a majority of human cancers, including in lung cancer. The subsequent expression analysis by qPCR and Western blot validated its overexpression in our collected NSCLC tissues. Importantly, its higher expression was clearly associated with advanced grade and lymph node metastasis status (T/N stage), strongly indicating its implication in NSCLC progression and metastasis. Regarding metastasis, another crucial piece of evidence is that we observed WT1 overexpression in high-metastatic 95D and L9981 cells compared with the corresponding low-metastatic 95 C and NL9980 cells, respectively. Since the relevance of WT1 in NSCLC metastasis remains unknown, our findings might provide novel clues. Kaplan-Meier plotter-derived survival analysis further showed that higher WT1 expression predicted shorter survival of lung cancer patients. Immune infiltration abundance analyses supported that patients with higher WT1 expression might benefit from immunotherapy. Therefore, these findings confirm the overexpression of WT1 in NSCLC, on the other hand, imply the association of WT1 with the metastasis of NSCLC cells, and suggest the potential of WT1 as a novel prognostic biomarker in NSCLC.

Network analysis of WT1 coexpressed genes indicated that WT1 was coexpressed with genes functionally involved in cell motility or aggressiveness of cancer cells, again suggesting its association with the metastasis of NSCLC cells. In fact, Wu et al. reported that WT1 promoted NSCLC cell invasion by inhibiting cadherin 1 (CDH1) transcription [38]. Through comprehensive in vitro and in vivo assays, we speculated that WT1 acted as a potent tumour promoter by facilitating NSCLC cell proliferation, survival, invasion and tumorigenesis. Our findings fully supported the previous conclusion and provided comprehensive and strong evidence.

MiRNAs are crucial modifiers of gene expression in lung cancer [39]. Previously, WT1 was identified as a target of miR-361 and facilitated cell growth, migration and invasion in NSCLC [40]. However, one specific mRNA can be targeted by multiple different miRNAs. One of the novel insights of our study is that WT1 could be directly targeted by miR-498-5p. MiR-498-5p is a double-edged miRNA possessing both tumour suppressing and tumour promoting capacities in different cancers. For instance, it facilitates cell proliferation, migration or invasion in prostate cancer [41], breast cancer [42] and retinoblastoma [43], whereas it does the opposite in ovarian cancer [44] and gastric cancer [45]. In lung cancer, several reports have recognized miR-498-5p as a tumour suppressor by targeting HMGA2 [22], tripartite motif containing 44 (TRIM44) [46] or forkhead box O3 (FOXO3) [47]. Herein, we provide another vital target of miR-498-5p, WT1. We also confirmed that miR-498-5p inhibited NSCLC progression and displayed lower expression in NSCLC tissues and high-metastatic cell lines. The inverse correlation between WT1 and miR-498-5p further verified their targeting relationship. Therefore, miR-498-5p downregulation governing WT1 overexpression is an important mechanism in NSCLC. Importantly, WT1 restoration attenuated the tumour suppressing effects of miR-498-5p in vitro and in vivo, once more illustrating the oncogenic activities of WT1 in NSCLC cells.

## Conclusion

To conclude, our findings revealed the tumour promoting potential of WT1 in NSCLC cells by facilitating cell proliferation, survival, invasion and tumorigenesis. We also identified miR-498-5p as a crucial upstream natural inhibitor of WT1 in NSCLC cells. In addition, higher WT1 levels might serve as a novel prognostic biomarker for lung cancer patients. Further mechanistic investigation of WT1 in transcription and signal transduction regulation will enhance our understanding of its complex roles in human cancers. Nevertheless, our findings shed light on the possibility of targeting WT1 in NSCLC therapy.

### Electronic supplementary material

Below is the link to the electronic supplementary material.

Supplementary Material 1

## Data Availability

All data used or analyzed during the current study are available from the corresponding author on reasonable request.

## Abbreviations

WT1: Wilms’ tumour gene 1
NSCLC: Non-small cell lung cancer
miRNAs: MicroRNAs
UTR: Untranslated region
DMEM: Dulbecco’s Modified Eagle Medium
FBS: Fetal bovine serum
qPCR: Quantitative PCR
SDS-PAGE: Sodium dodecyl sulfate polyacrylamide gel electrophoresis
MMP2: Metallopeptidase 2
CCK8: Cell Counting Kit-8
PBS: Phosphate buffer saline
NC: Negative control
LUAD: Lung adenocarcinoma
LUSC: Lung squamous cell carcinoma
LCLC: Large cell lung carcinoma
TME: Tumour immune microenvironment
GO: Gene ontology
ECM: Extracellular matrix
siRNAs: Small interfering RNAs
