# Correction: Wilms’ tumour gene 1 (WT1) enhances non-small cell lung cancer malignancy and is inhibited by microRNA-498-5p

**DOI:** 10.1186/s12885-023-11399-9

**Published:** 2023-09-27

**Authors:** Xuebing Li, Wenzhe An, Hongli Pan, Yaguang Fan, Hua Huang, Yixuan Wang, Wang Shen, Lingling Zu, Fanrong Meng, Xuexia Zhou

**Affiliations:** 1 Tianjin Key Laboratory of Lung Cancer Metastasis and Tumor Microenvironment, Tianjin Lung Cancer Institute, Department of Lung Cancer Surgery, Tianjin Medical University General Hospital, Tianjin, China; 2 Department of Neuropathology, Tianjin Key Laboratory of Injuries, Variations and Regeneration of the Nervous System, Key Laboratory of Post-trauma Neuro-repair and Regeneration in Central Nervous System of Education Ministry, Tianjin Neurological Institute, Tianjin Medical University General Hospital, Tianjin, China; 3 West China Hospital, Sichuan Lung Cancer Center, Sichuan Lung Cancer Institute, Sichuan University, Chengdu, China; 4 Tianjin Prenatal Diagnostic Center, Obstetrics and Gynecology Department, Tianjin Medical University General Hospital, Tianjin, China

**Correction:** ***BMC Cancer* 23, 824 (2023)**

10.1186/s12885-023-11295-2

Following publication of the original article [1], it was reported that there was an error in the PDF version of the published article. Figures 5 and 7 were missing and Figs. 6 and 8 were placed out of order.

The PDF version of the original article has been corrected to rectify these errors.
