# Supplementary material for: Wilms’ tumour gene 1 (WT1) enhances non-small cell lung cancer malignancy and is inhibited by microRNA-498-5p
Source: BMC Cancer. 2023 Sep 4;23:824. doi: 10.1186/s12885-023-11295-2 (PMC10476375; doi:10.1186/s12885-023-11295-2)
Supplement: Supplementary file 1 — Supplementary Material 1 [file 12885_2023_11295_MOESM1_ESM.pptx]

## Slide 1
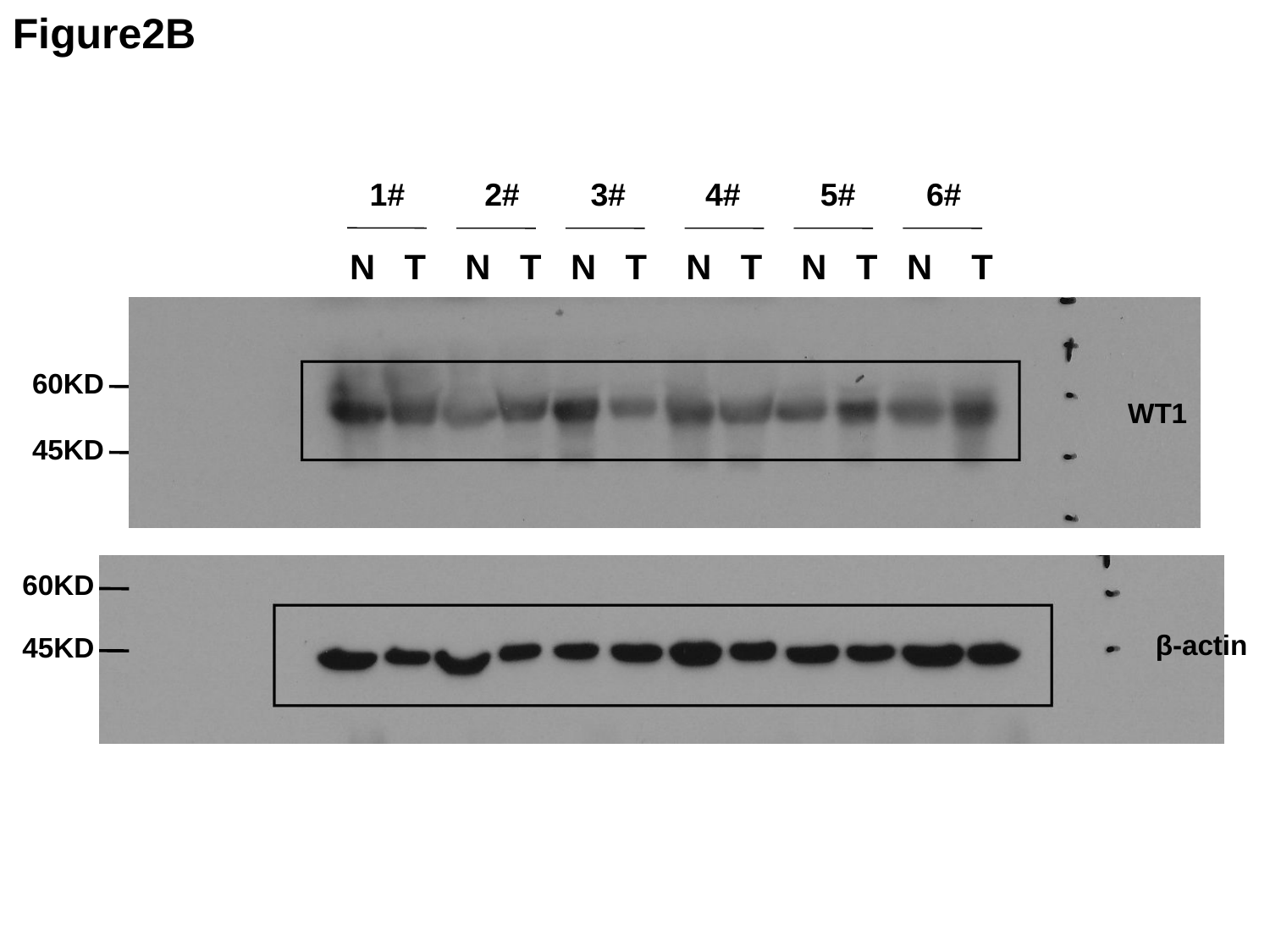

Figure2B
1# 2# 3# 4# 5# 6#
N T N T N T N T N T N T
60KD
WT1
45KD
60KD
β-actin
45KD

## Slide 2
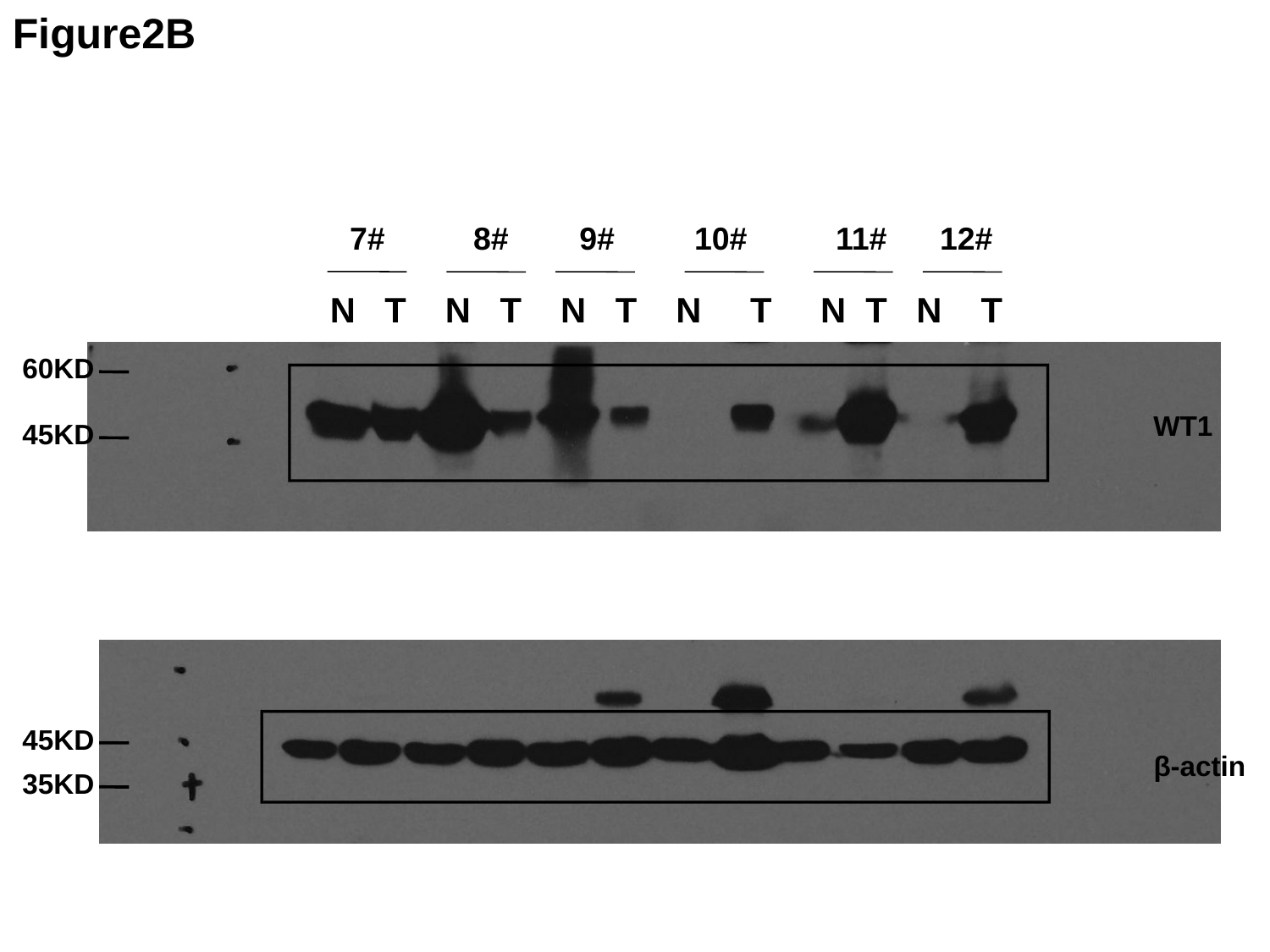

Figure2B
7# 8# 9# 10# 11# 12#
N T N T N T N T N T N T
60KD
WT1
45KD
45KD
β-actin
35KD

## Slide 3
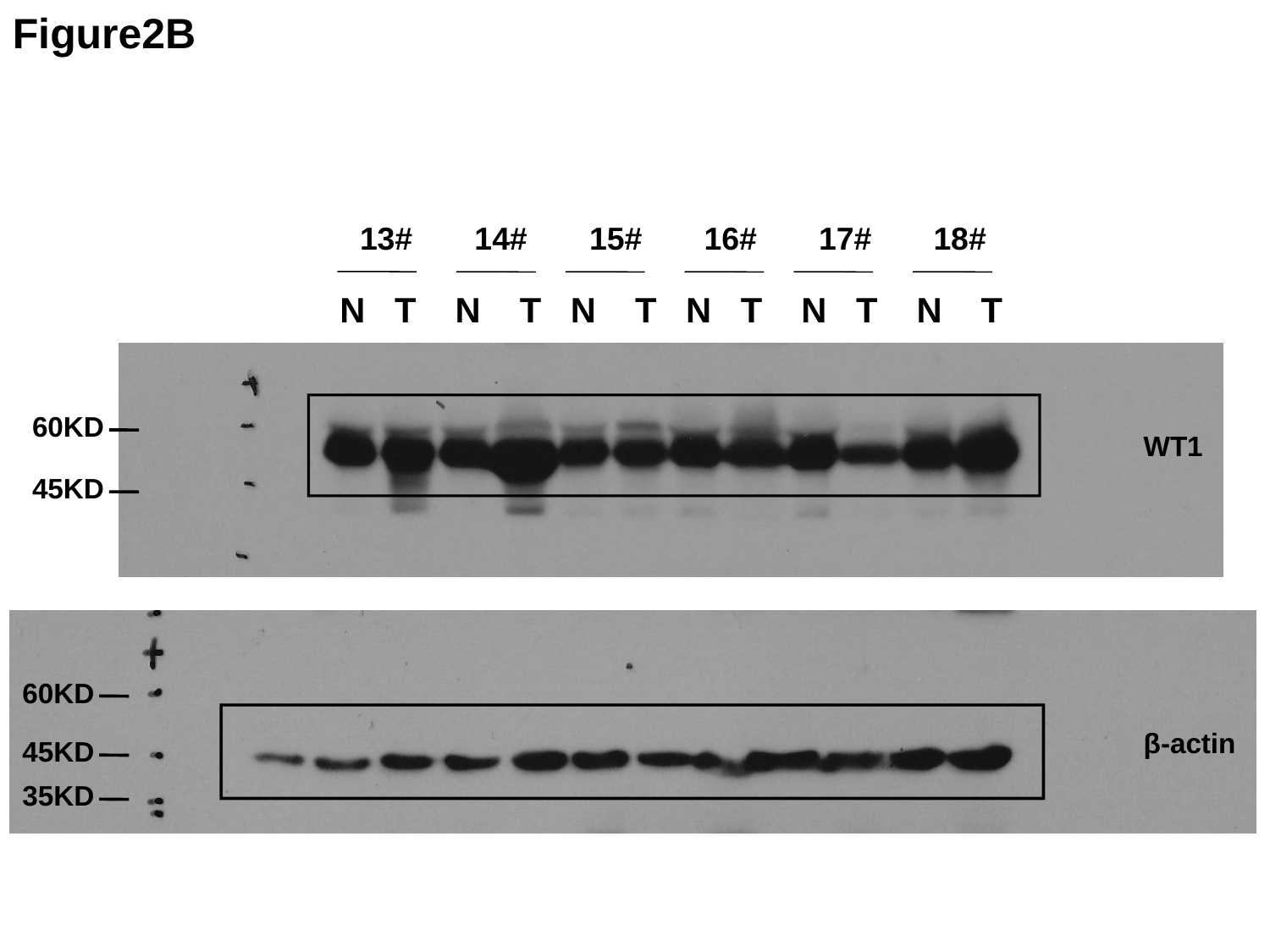

Figure2B
13# 14# 15# 16# 17# 18#
N T N T N T N T N T N T
60KD
WT1
45KD
60KD
β-actin
45KD
35KD

## Slide 4
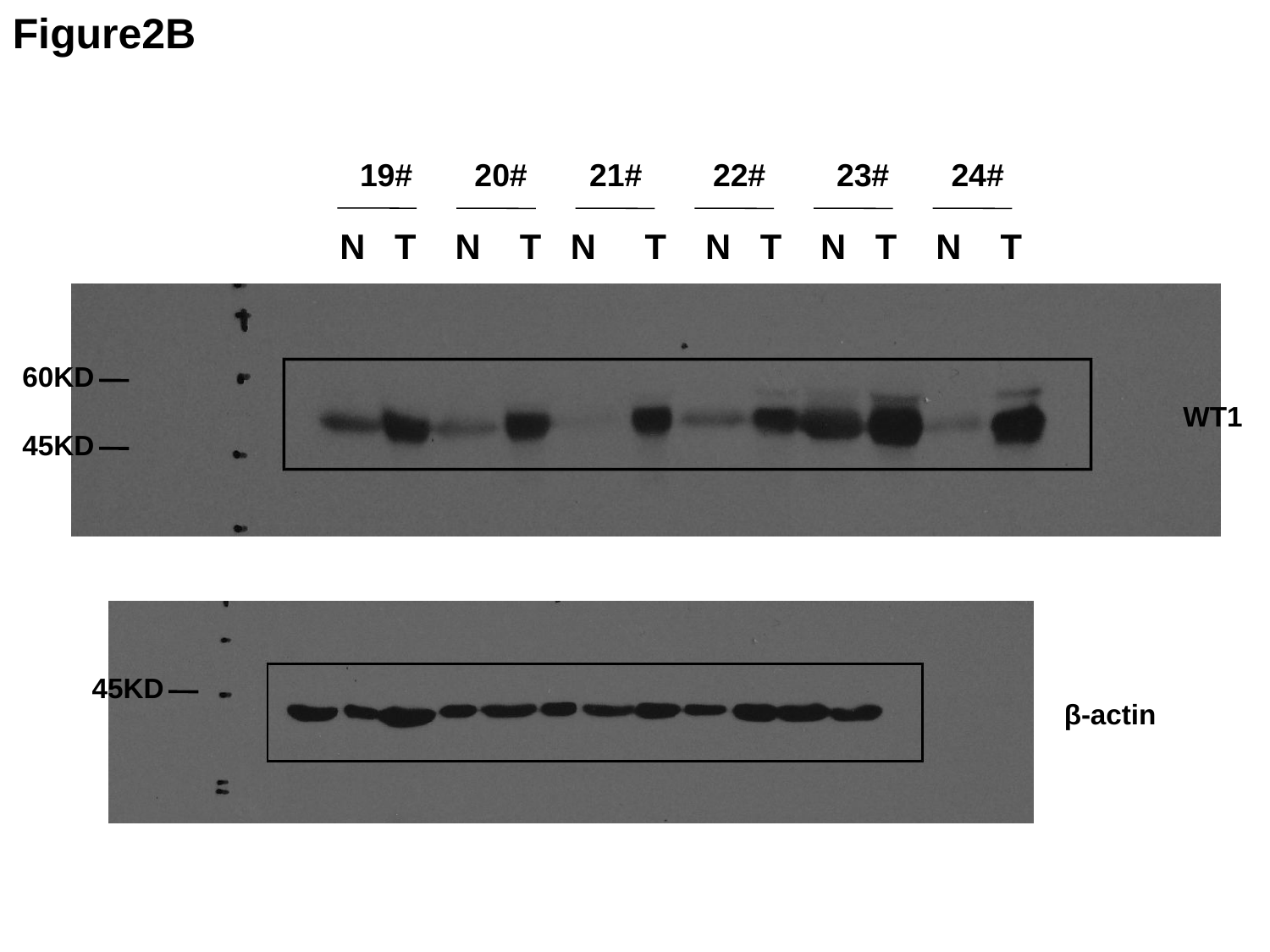

Figure2B
19# 20# 21# 22# 23# 24#
N T N T N T N T N T N T
60KD
WT1
45KD
45KD
β-actin

## Slide 5
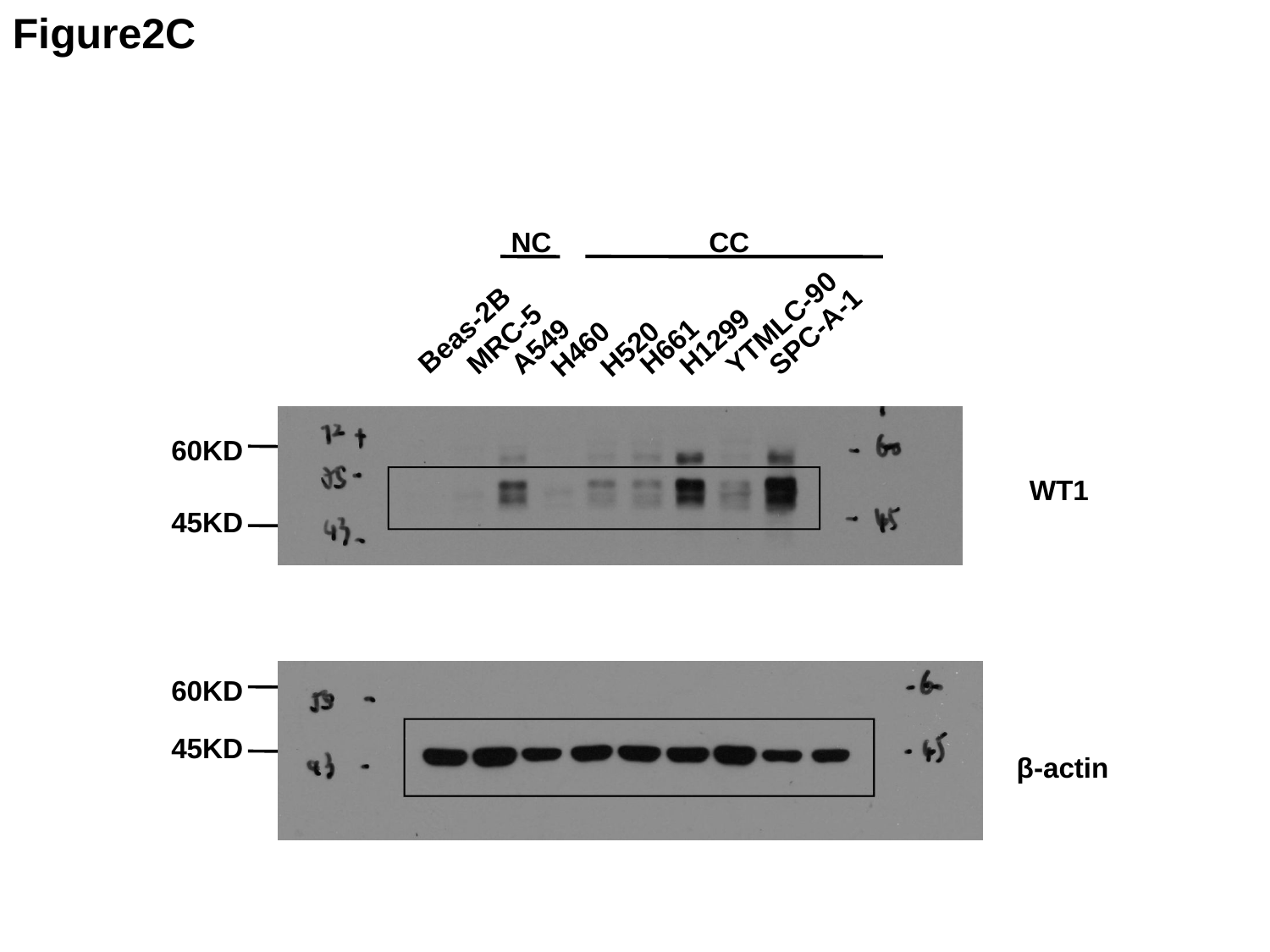

Figure2C
CC
NC
YTMLC-90
SPC-A-1
Beas-2B
MRC-5
H1299
A549
H661
H460
H520
60KD
WT1
45KD
60KD
45KD
β-actin

## Slide 6
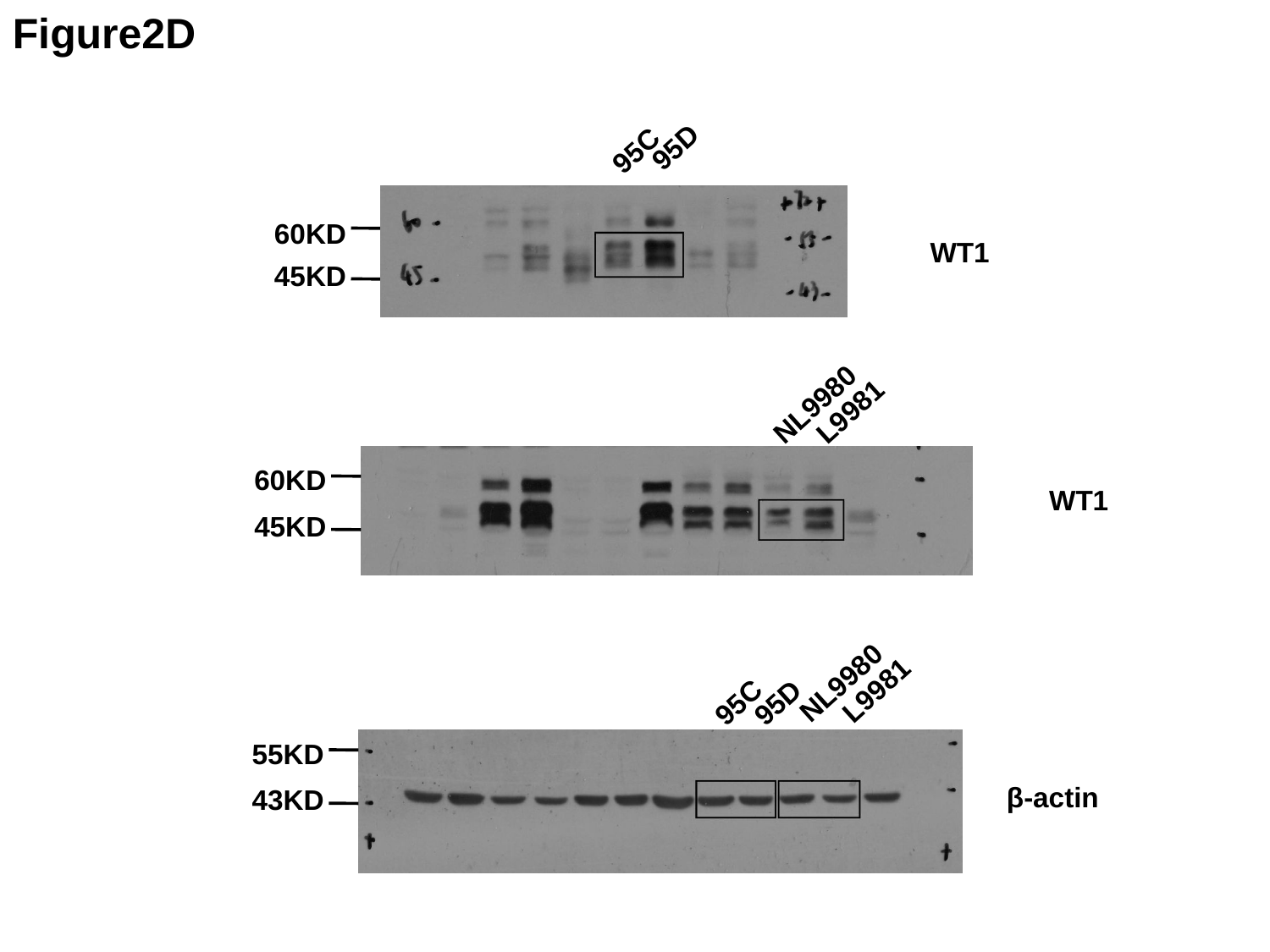

Figure2D
95C
95D
60KD
WT1
45KD
NL9980
L9981
60KD
WT1
45KD
NL9980
L9981
95C
95D
55KD
β-actin
43KD

## Slide 7
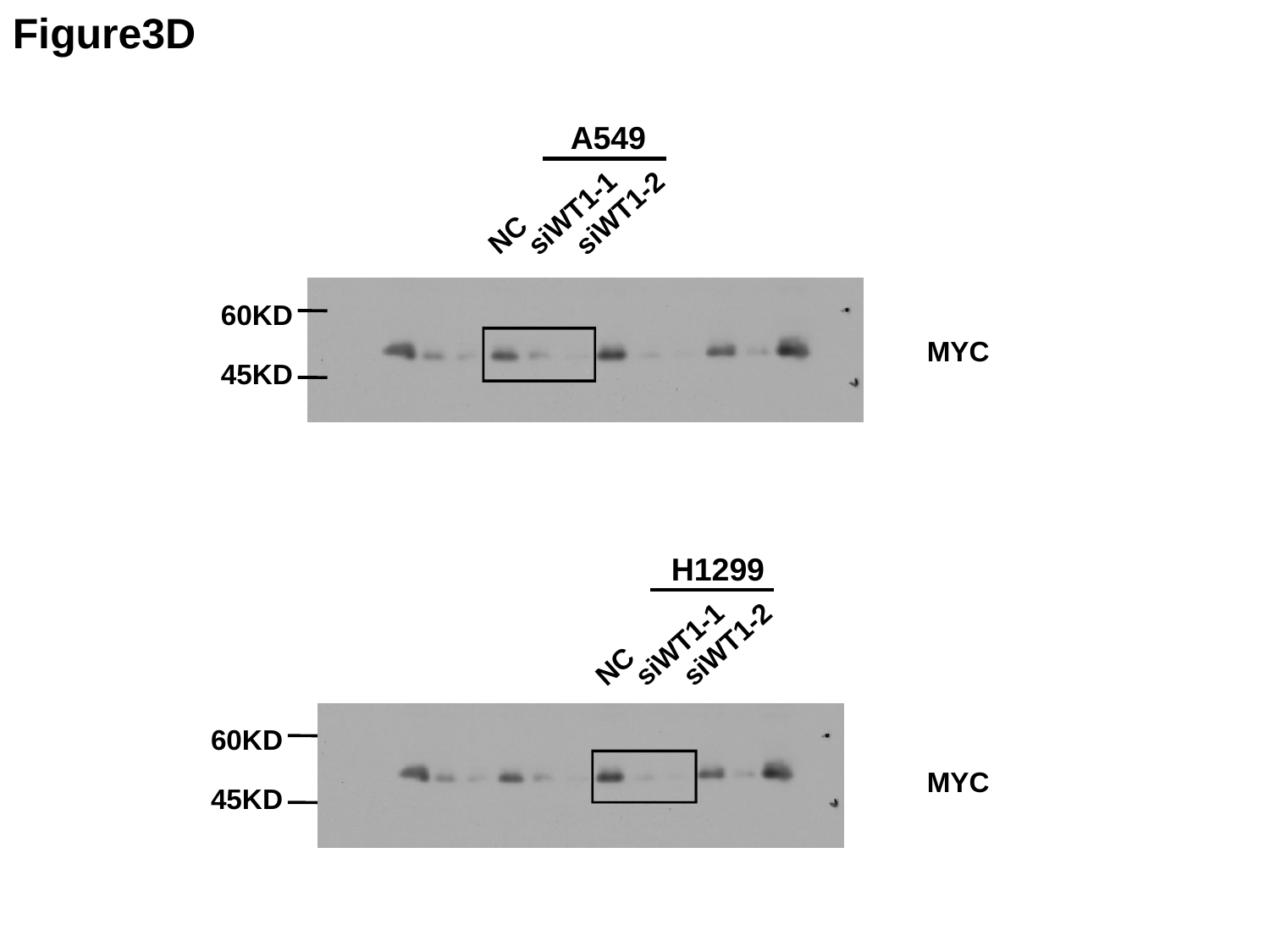

Figure3D
A549
siWT1-1
siWT1-2
NC
60KD
MYC
45KD
H1299
siWT1-1
siWT1-2
NC
60KD
MYC
45KD

## Slide 8
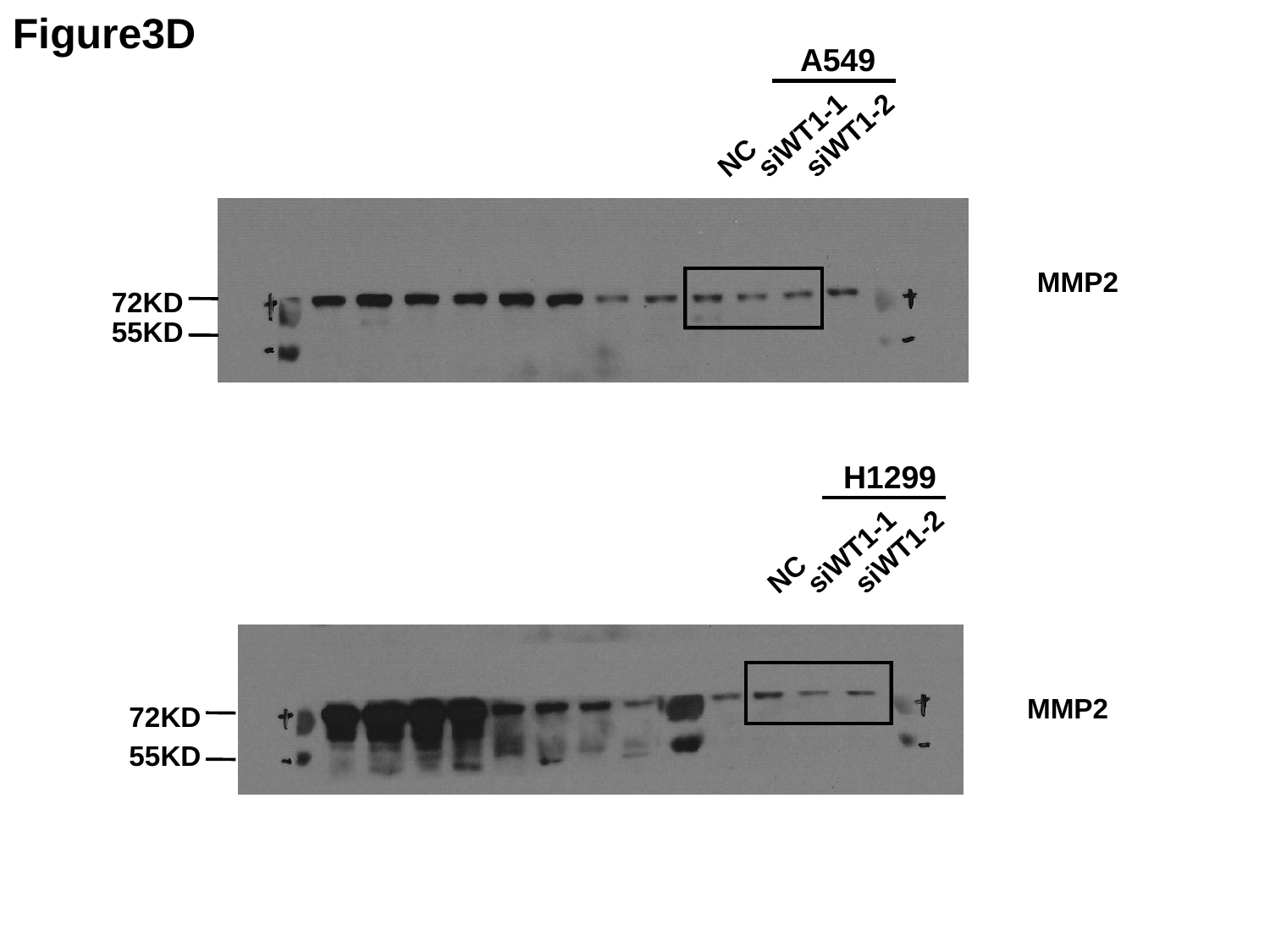

Figure3D
A549
siWT1-1
siWT1-2
NC
MMP2
72KD
55KD
H1299
siWT1-1
siWT1-2
NC
MMP2
72KD
55KD

## Slide 9
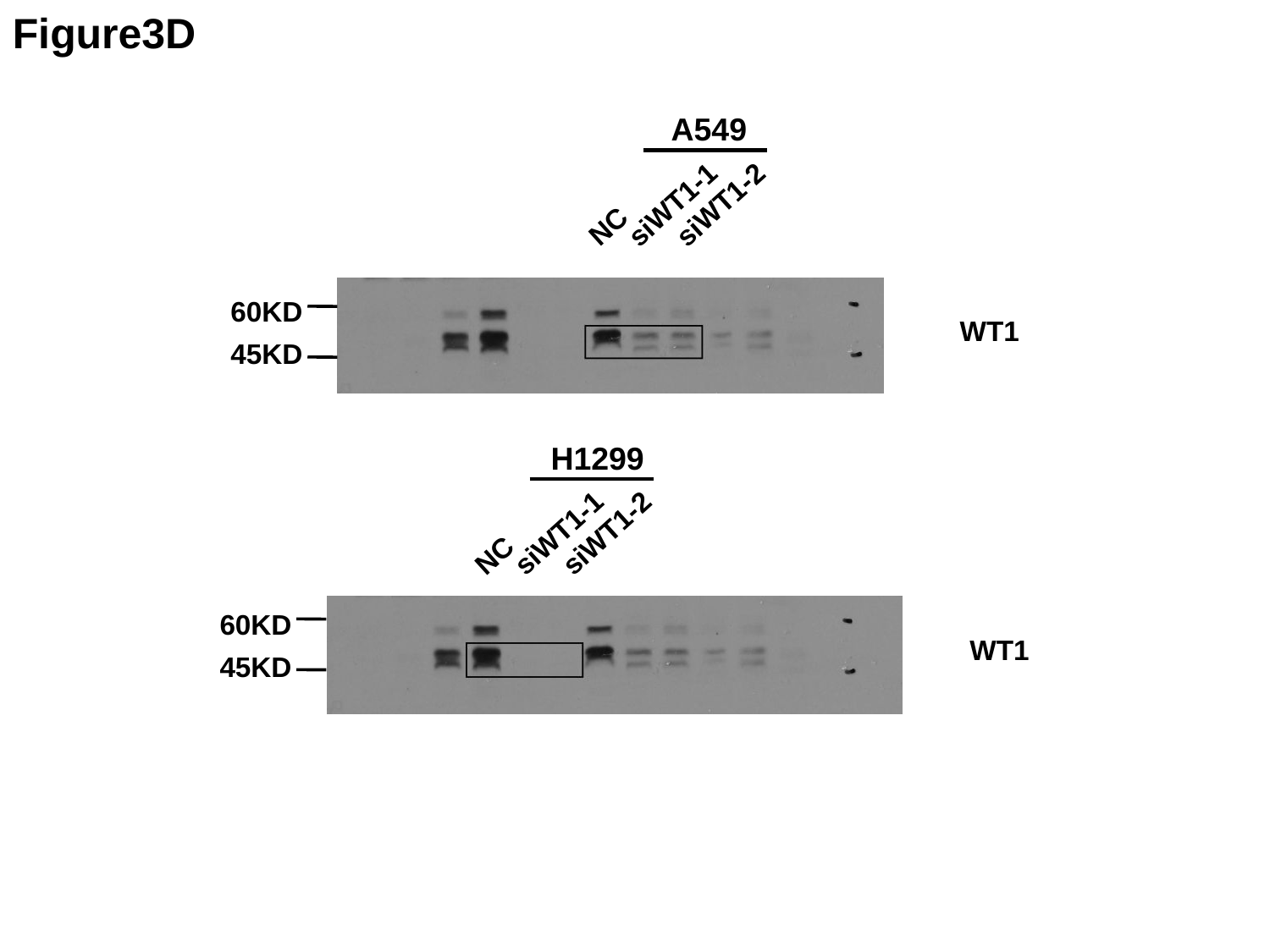

Figure3D
A549
siWT1-1
siWT1-2
NC
60KD
WT1
45KD
H1299
siWT1-1
siWT1-2
NC
60KD
WT1
45KD

## Slide 10
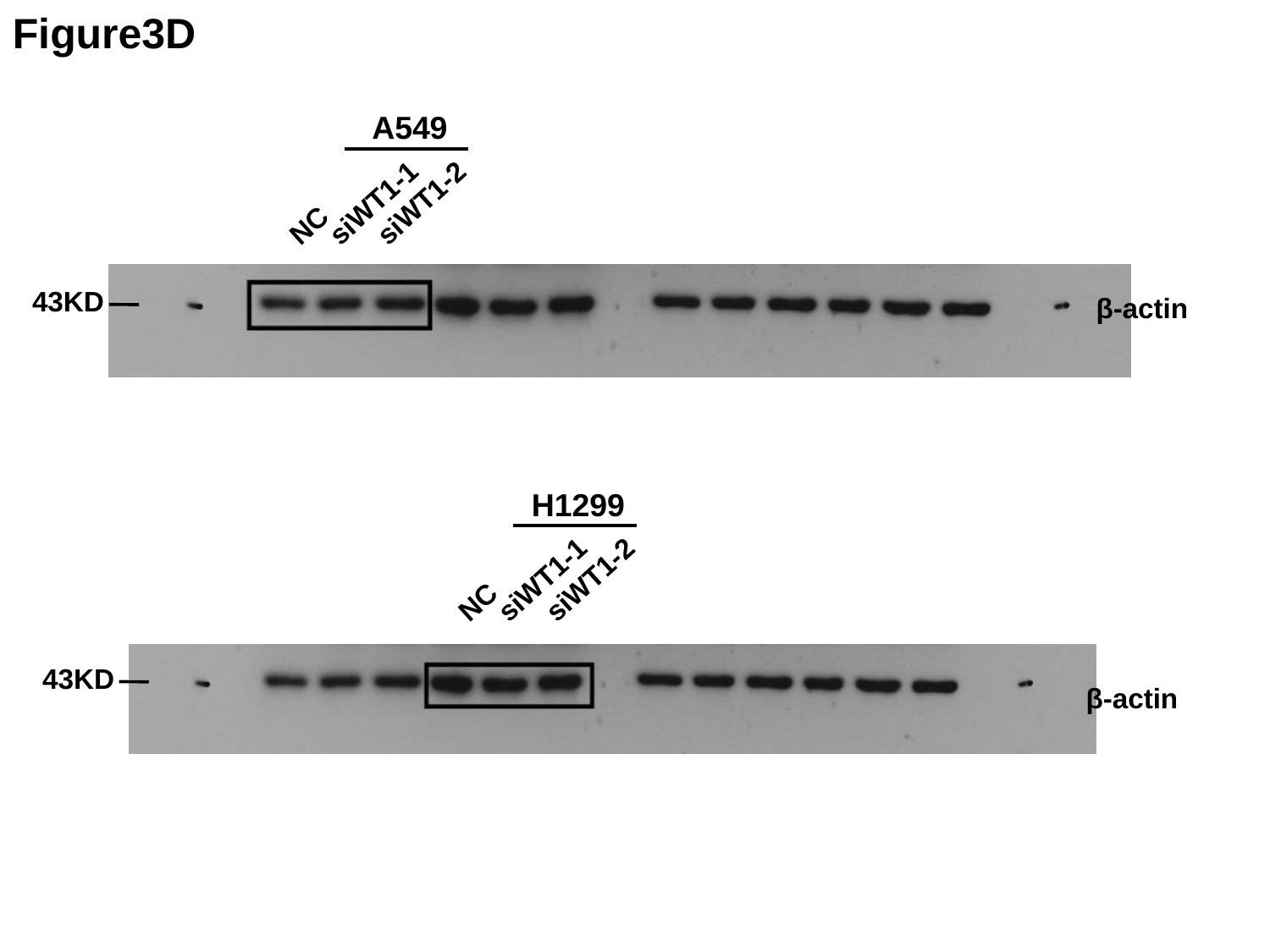

Figure3D
A549
siWT1-1
siWT1-2
NC
43KD
β-actin
H1299
siWT1-1
siWT1-2
NC
43KD
β-actin

## Slide 11
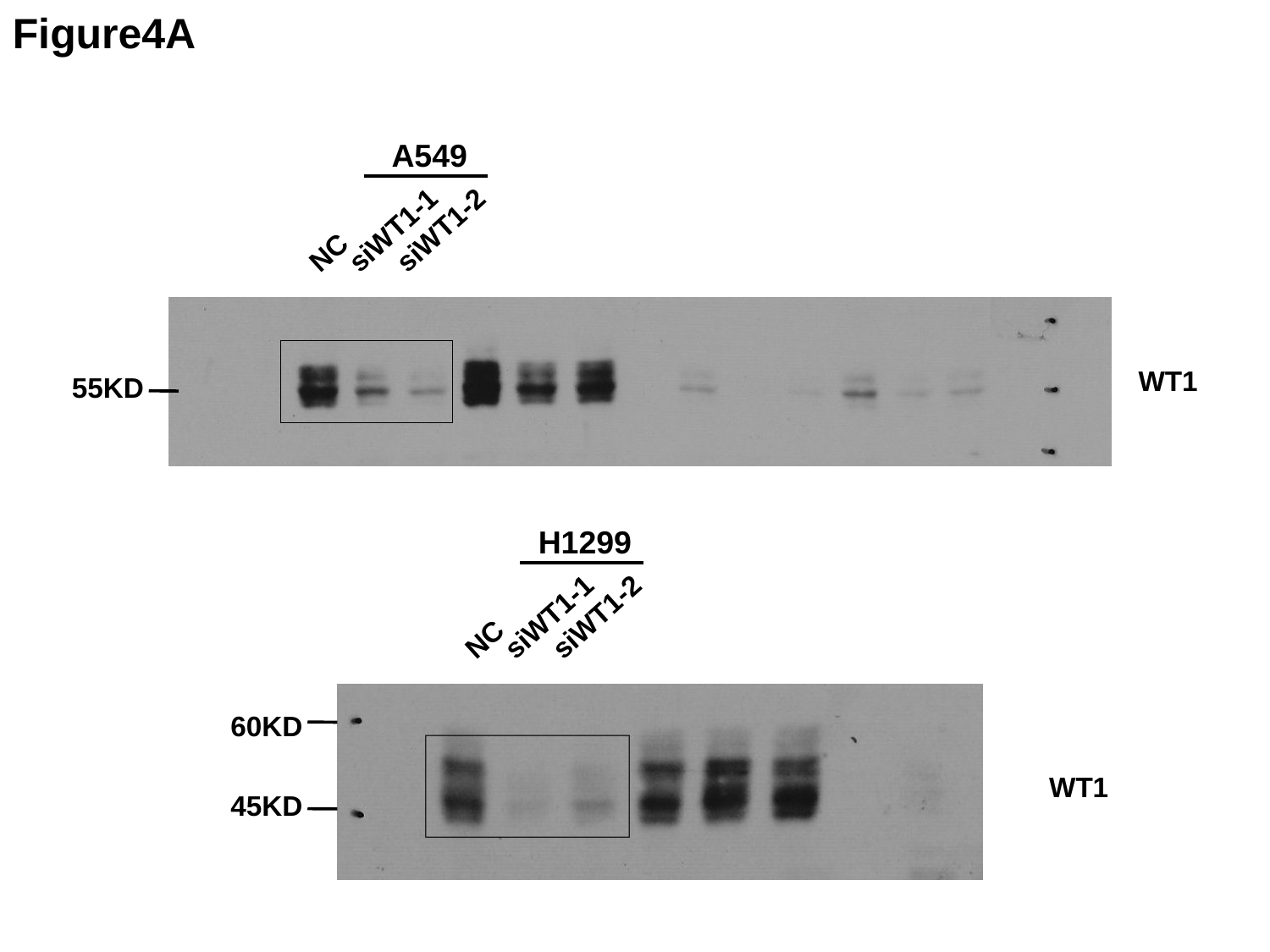

Figure4A
A549
siWT1-1
siWT1-2
NC
WT1
55KD
H1299
siWT1-1
siWT1-2
NC
60KD
WT1
45KD

## Slide 12
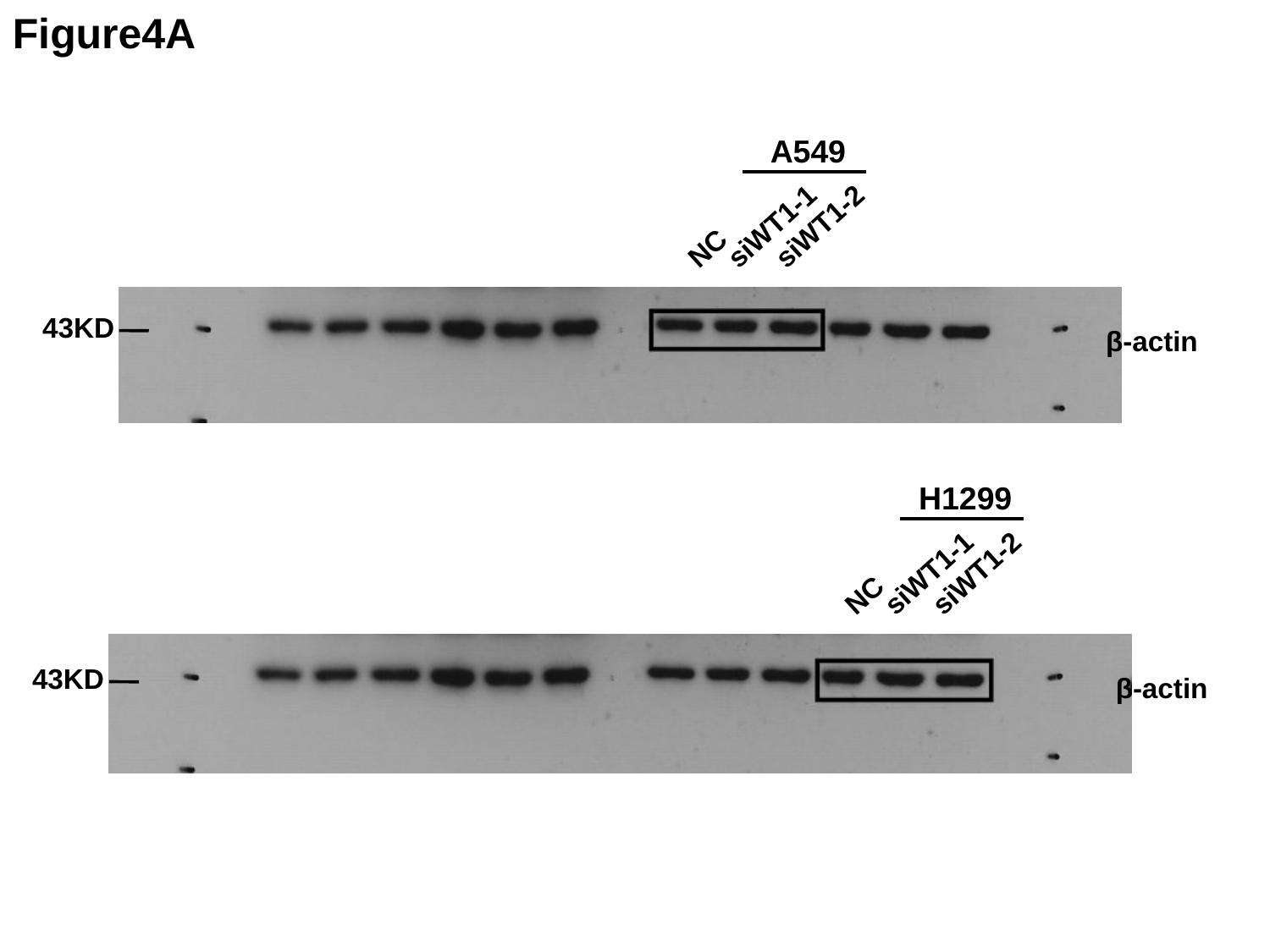

Figure4A
A549
siWT1-1
siWT1-2
NC
43KD
β-actin
H1299
siWT1-1
siWT1-2
NC
43KD
β-actin

## Slide 13
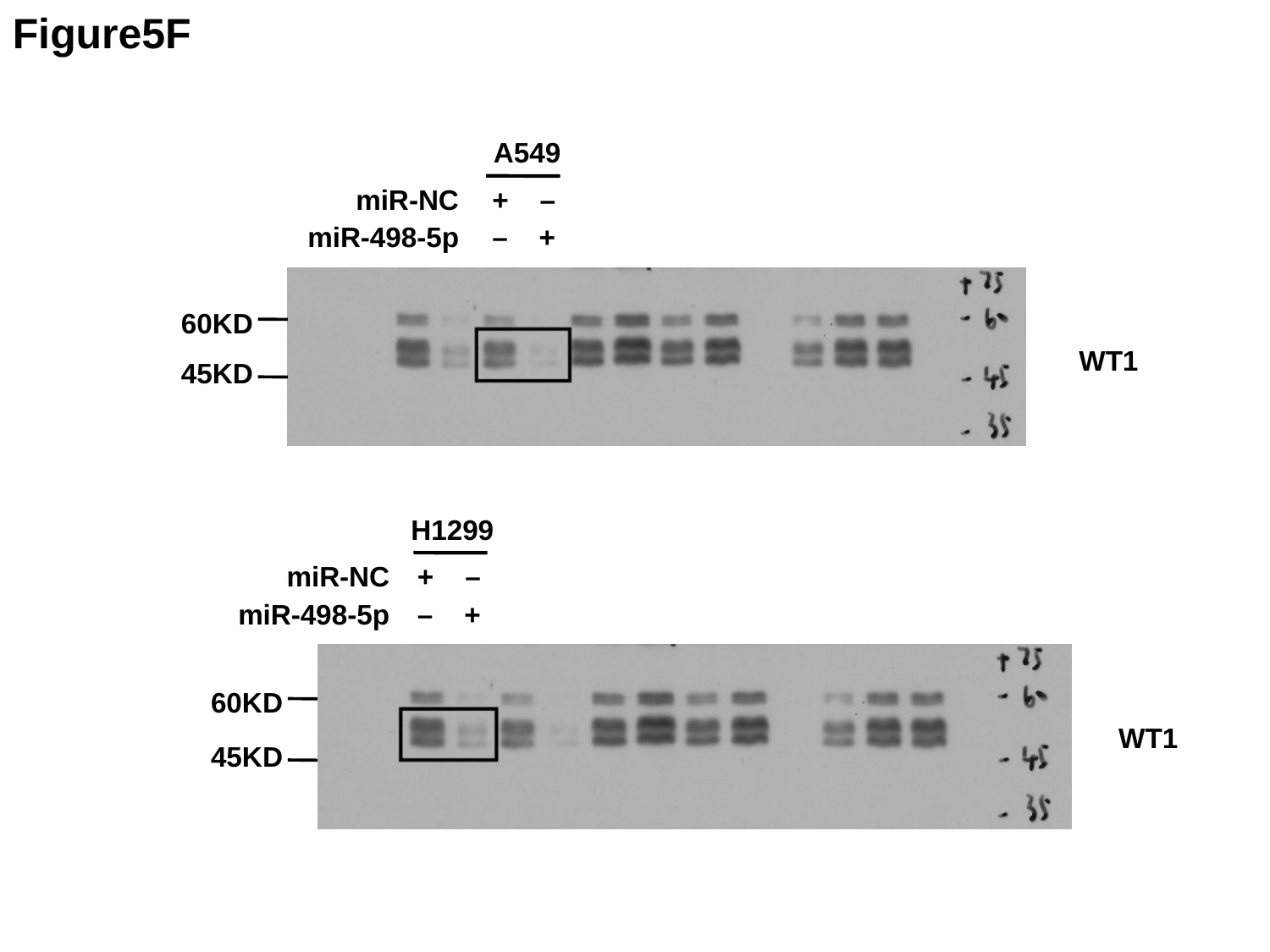

Figure5F
A549
miR-NC
+ –
miR-498-5p
– +
60KD
WT1
45KD
H1299
miR-NC
+ –
miR-498-5p
– +
60KD
WT1
45KD

## Slide 14
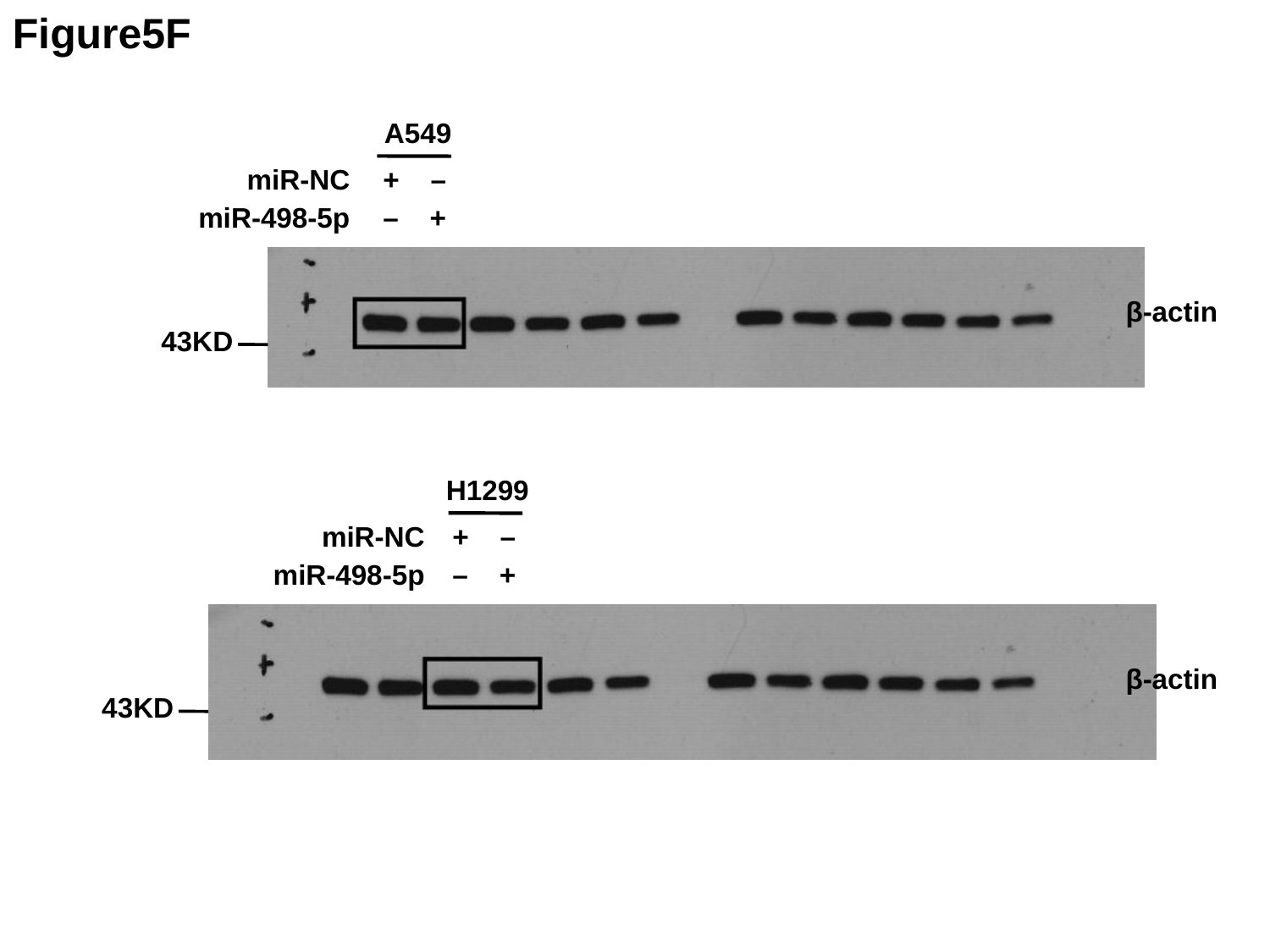

Figure5F
A549
miR-NC
+ –
miR-498-5p
– +
β-actin
43KD
H1299
miR-NC
+ –
miR-498-5p
– +
β-actin
43KD

## Slide 15
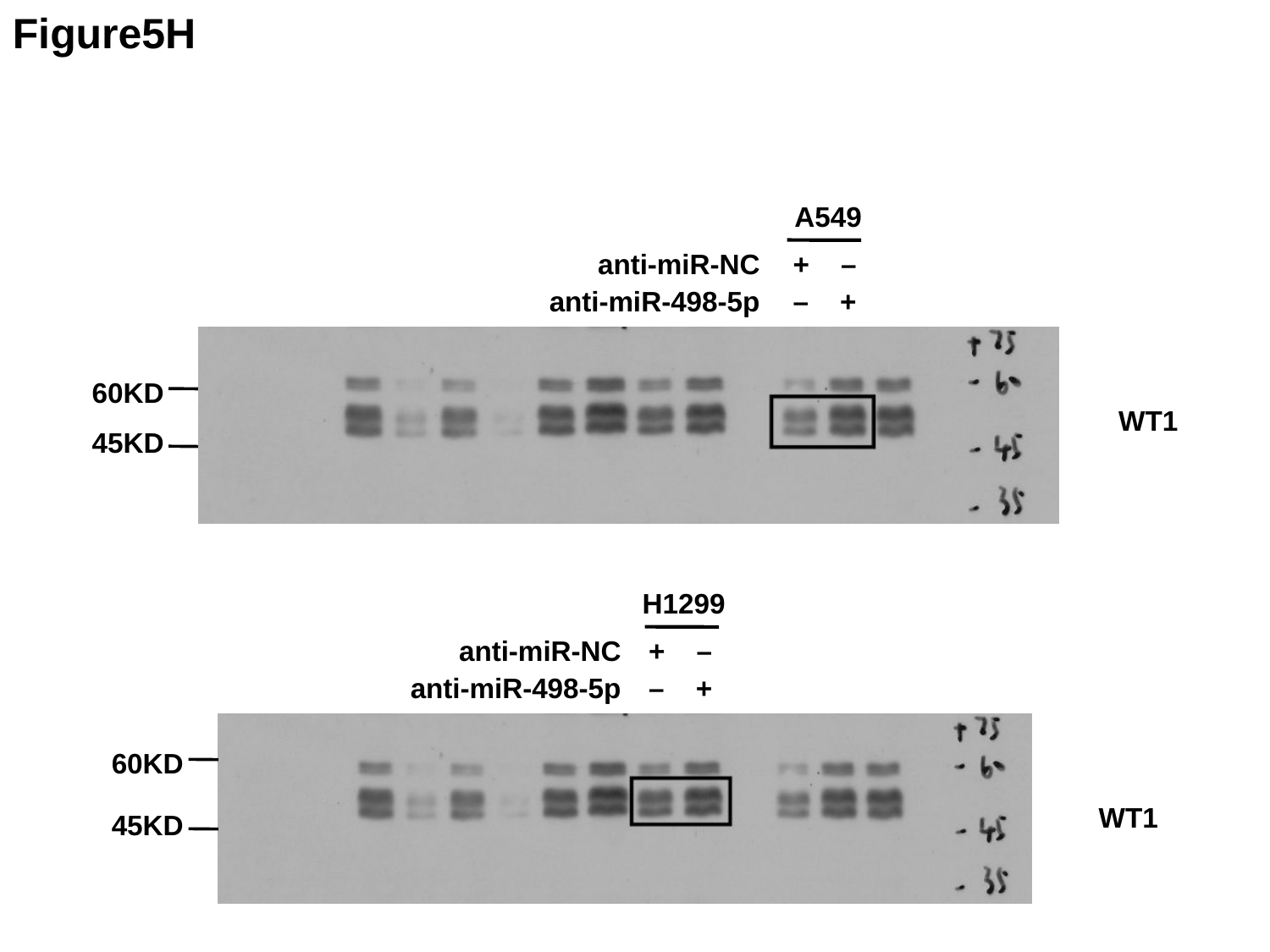

Figure5H
A549
anti-miR-NC
+ –
anti-miR-498-5p
– +
60KD
WT1
45KD
H1299
anti-miR-NC
+ –
anti-miR-498-5p
– +
60KD
WT1
45KD

## Slide 16
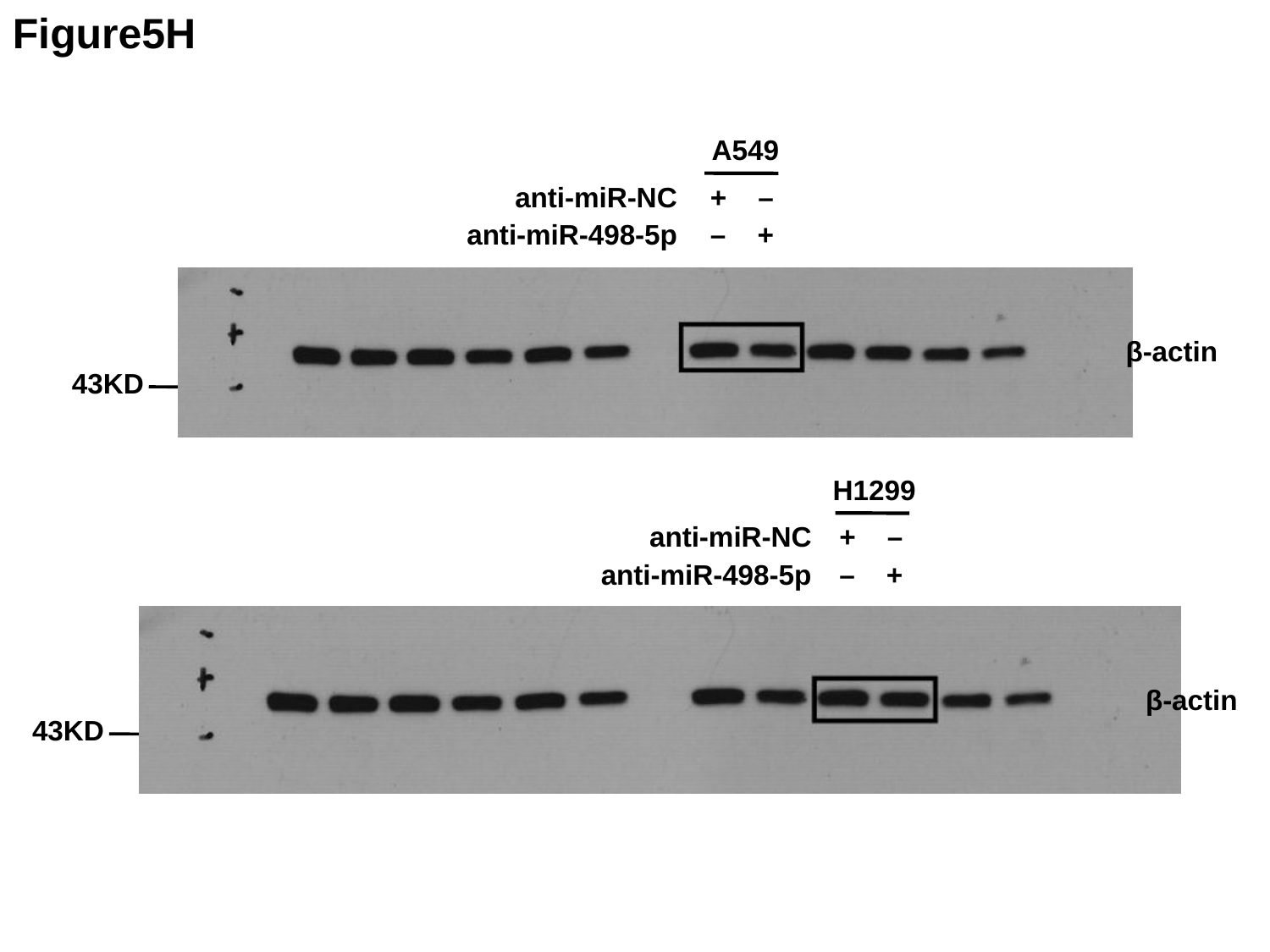

Figure5H
A549
anti-miR-NC
+ –
anti-miR-498-5p
– +
β-actin
43KD
H1299
anti-miR-NC
+ –
anti-miR-498-5p
– +
β-actin
43KD

## Slide 17
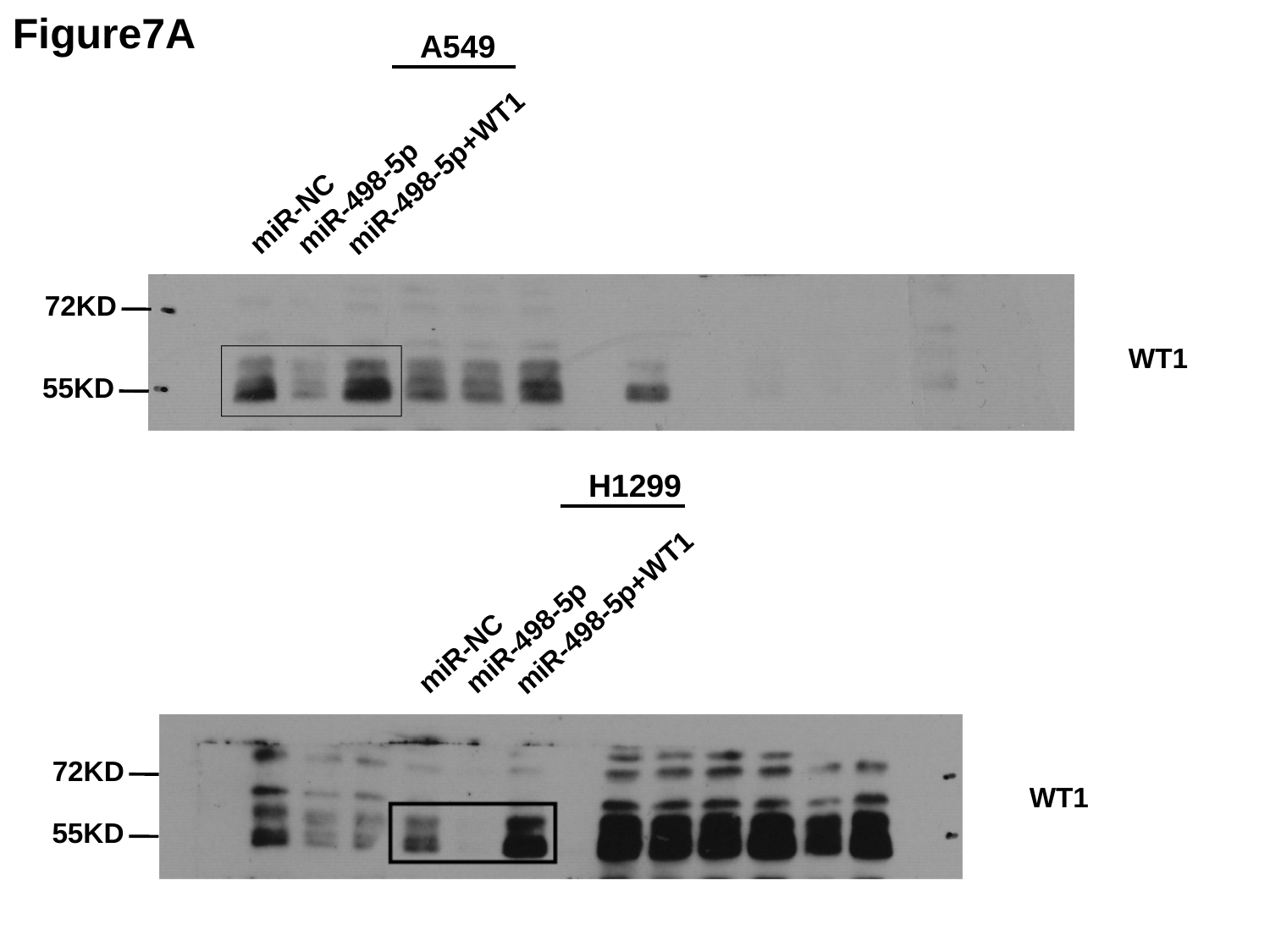

Figure7A
A549
miR-498-5p+WT1
miR-498-5p
miR-NC
72KD
WT1
55KD
H1299
miR-498-5p+WT1
miR-498-5p
miR-NC
72KD
WT1
55KD

## Slide 18
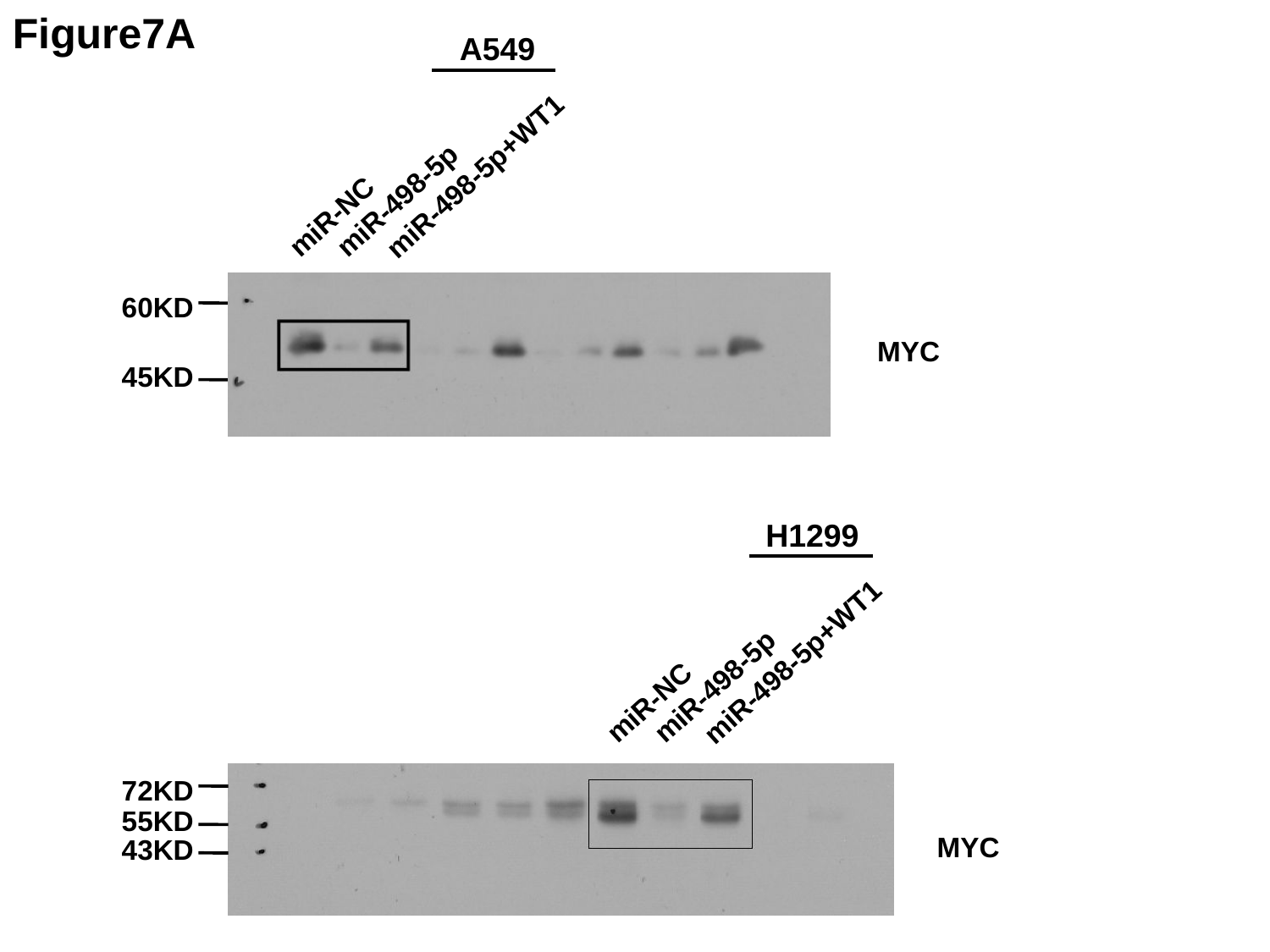

Figure7A
A549
miR-498-5p+WT1
miR-498-5p
miR-NC
60KD
MYC
45KD
H1299
miR-498-5p+WT1
miR-498-5p
miR-NC
72KD
55KD
MYC
43KD

## Slide 19
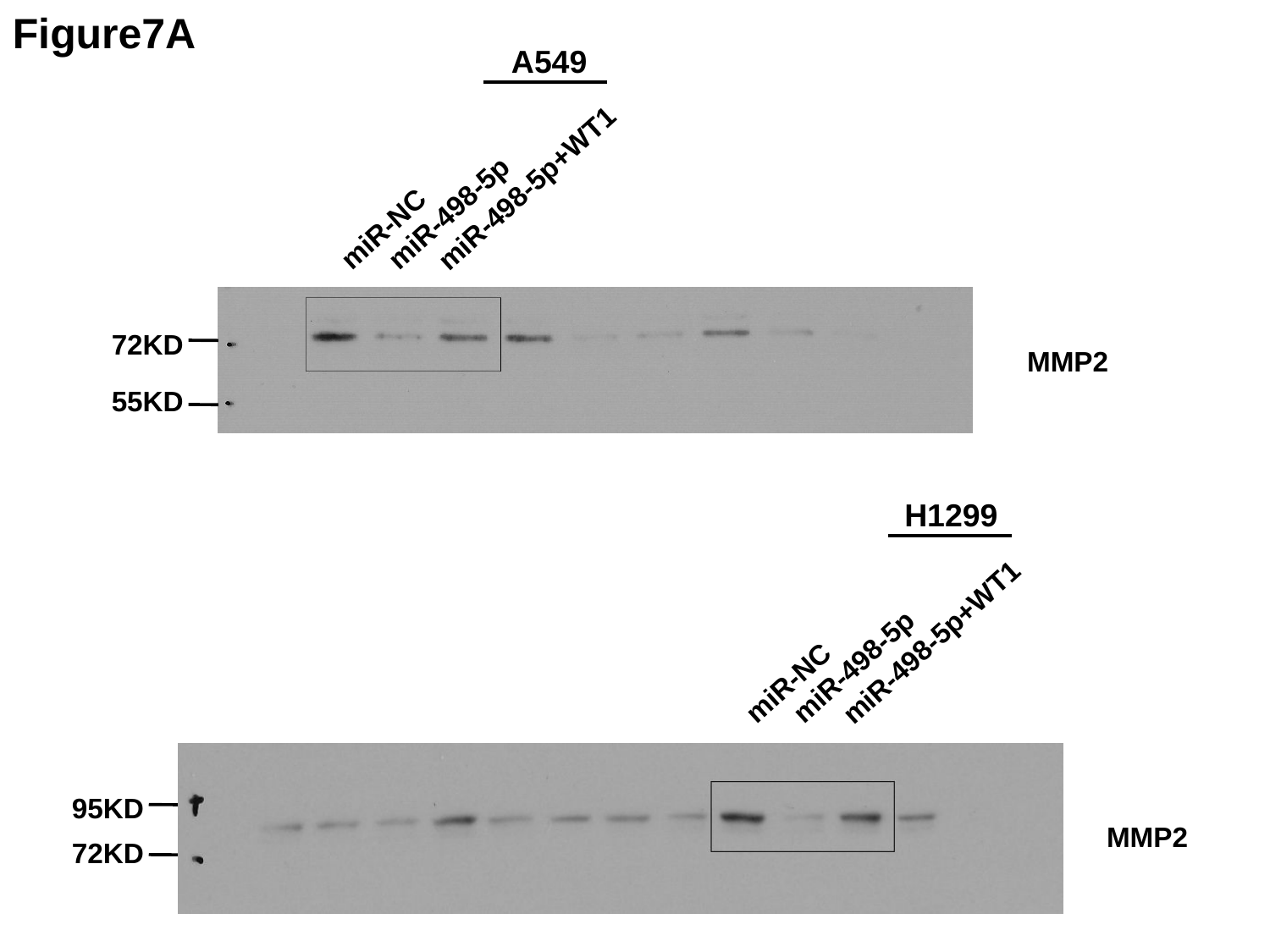

Figure7A
A549
miR-498-5p+WT1
miR-498-5p
miR-NC
72KD
MMP2
55KD
H1299
miR-498-5p+WT1
miR-498-5p
miR-NC
95KD
MMP2
72KD

## Slide 20
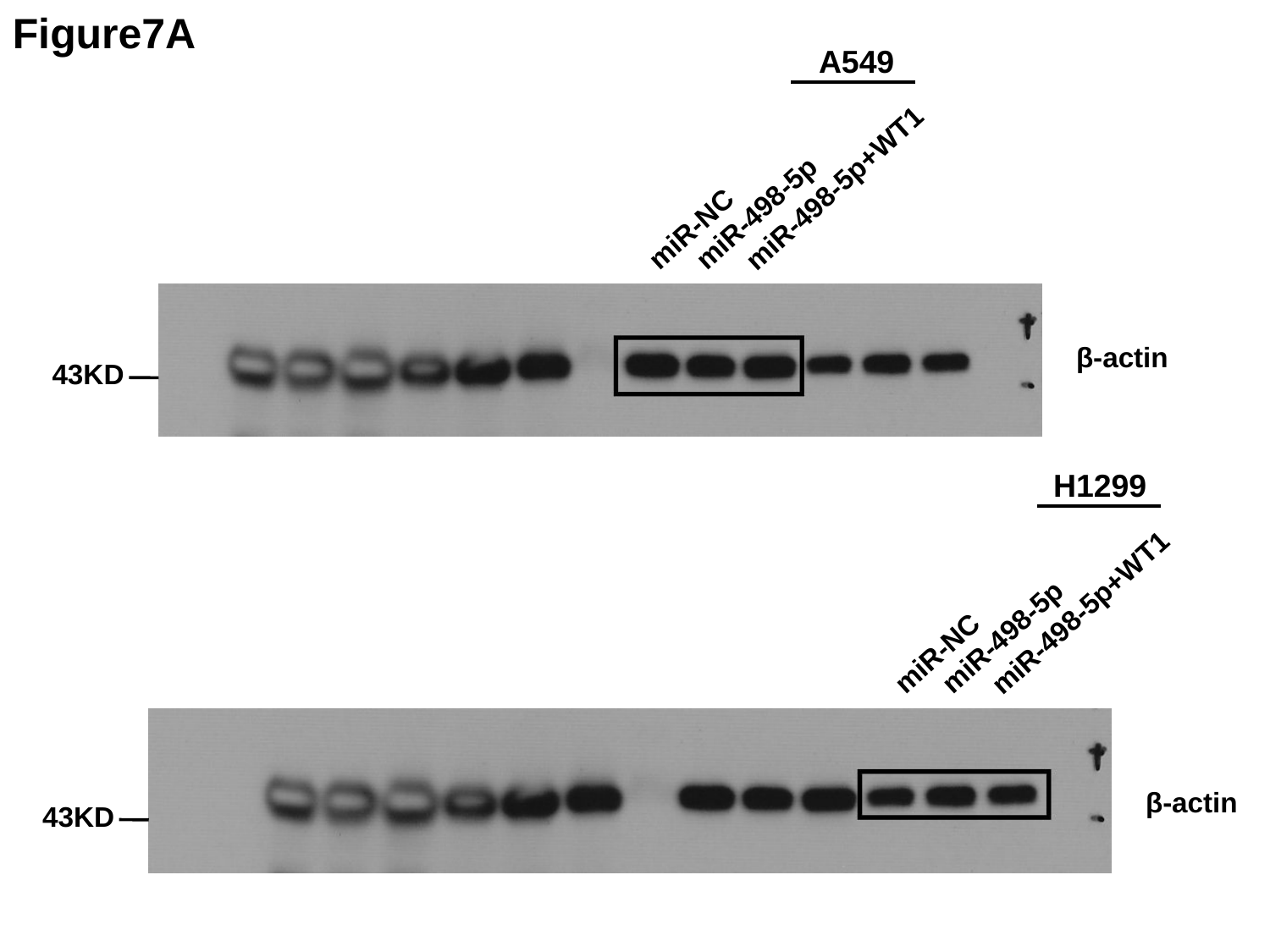

Figure7A
A549
miR-498-5p+WT1
miR-498-5p
miR-NC
β-actin
43KD
H1299
miR-498-5p+WT1
miR-498-5p
miR-NC
β-actin
43KD
